# Supplementary material for: Nanobody-based canine PD-L1-targeting immune checkpoint inhibitors for cancer therapy in dogs
Source: Mol Ther Oncol. 2025 Sep 8;33(3):201036. doi: 10.1016/j.omton.2025.201036 (PMC12587322; doi:10.1016/j.omton.2025.201036)
Supplement: Document S1. Figures S1–S6 and Tables S1–S3 [file mmc1.pdf]

OMTON, Volume *33*

Supplemental information

Nanobody-based canine PD-L1-targeting immune checkpoint inhibitors for cancer therapy in dogs

Morgane Di Palma Subran, Marianne Wyss, Betül Taskoparan, Mathischan Maheswaran, Johannes vom Berg, Philippe Plattet, and Patrick Chames

SUPPLEMENTAL MATERIALS AND METHODS

NK cells isolation

Primary human NK cells were isolated from peripheral blood of healthy donors obtained from the French National Blood Bank (Etablissement Français du Sang) using ethically approved procedures. The NK isolation was performed by negative selection using the MACSxpress Whole Blood human NK cell isolation kit (cat. no. 130–098–185, Miltenyi Biotec, Bergisch Gladbach, Germany), according to the manufacturer protocol. Cells were stored in RPMI 1640 medium (Gibco, Life Technologies, Carlsbad, CA, USA) complemented with 10% fetal bovine serum (FBS, Gibco, Life Technologies, Carlsbad, CA, USA) at 37 °C and used in the following 24 h.

The purity of the NK cells was determined by flow cytometry. Isolated primary NK Cells were plated at 200'000 cells per well in 96-well plates and incubated with phenotyping antibodies: anti-CD3-vioblue (cat. 130-094-363), anti-CD56-APC (cat. 130-113-310) and anti-CD16-FITC (cat. 130-113-392) (all from Miltenyi Biotec, Bergisch Gladbach, Germany). After washing, cells were analyzed using a MACSQuant X Fast cytometer (Miltenyi Biotec, Bergisch Gladbach, Germany) and affinity was measured as median of fluorescence (MFI).

Luminescence assay for ADCC measurements

Target cells were plated at 5000 cells/well in 96-well plates (cat. 655083, Greiner, Kremsmünster, Austria) and incubated at 37 °C and 5% CO2 for 6h. Serial dilutions of Nb constructs were added, and cells were incubated for 30 min. Isolated primary NK cells were added (effector:target ratio of 5:1). Cells were further incubated overnight, and the supernatant was rinsed. The cell viability was measured using CellTiter-Glo reagent (Promega, Madison, WI, USA), according to the manufacturer's instructions. The assay reports the amount of ATP from living cells in the sample. The total lysis was determined as followed:

$$\text{Equation S1: } \%Total\ Lysis = 100 \times (1 - \frac{Normalized\ cell\ index_{cells\ alone}}{Normalized\ cell\ Indes_{cells+PBMC+Ab}})$$

SUPPLEMENTAL TABLES

Table S1: Production of the nanobody constructs

Binder	Yield (mg/L)
34-41-hFc IgG1	**248.5**
41-34-hFc IgG1	**252.0**
34-41-dFc IgGB	**161.0**
41-34-dFc IgGB	**161.0**
34-41-dFc IgGA	**37.0**
41-34-dFc IgGA	**48.0**
34-41	**179.5**
41-34	**116.0**
34-hFc IgG1	**600.0**
34-dFc IgGB	**307.0**
34-dFc IgGA	**35.6**
41-hFc IgG1	**849.0**
41-dFc IgGB	**453.0**
41-dFc IgGA	**17.0**

Table S2: Affinity binding of the nanobody constructs by BLI

Binder	KD (M)	KD Error	ka (1/Ms)	ka2	ka Error	ka2 Error	kdis (1/s)	kdis2	kdis Error	kdis2 Error	Full X^2	Full R^2
34-41	**2.21E-08**	4.78E-10	**3.33E05**	9.97E00	**1.98E03**	2.76E00	**7.35E-03**	4.48E-02	**1.53E-04**	1.21E-02	0.05	0.99
34-41	**5.38E-09**	9.44E-11	**2.20E05**	4.72E-02	**1.42E03**	2.31E-03	**1.18E-03**	<1,0E-07	**1.93E-05**	<1,0E-07	0.04	0.99
34-41	**2.51E-08**	7.20E-10	**3.49E05**	2.83E00	**2.34E03**	5.07E-01	**8.76E-03**	1.53E-02	**2.44E-04**	2.54E-03	0.07	0.99
34-41-dFc IgGA	**1.86E-09**	4.99E-11	**2.30E05**	8.46E-02	**1.71E03**	3.83E-03	**4.29E-04**	1.83E-03	**1.10E-05**	3.79E-05	0.17	0.99
34-41-dFc IgGA	**7.16E-09**	7.87E-11	**2.05E05**	3.47E00	**6.39E02**	1.00E00	**1.46E-03**	1.79E-02	**1.54E-05**	4.92E-03	0.15	0.99
34-41-dFc IgGA	**4.95E-09**	6.75E-11	**2.42E05**	4.32E00	**1.20E03**	2.13E-01	**1.20E-03**	2.64E-02	**1.52E-05**	1.44E-03	0.32	0.99
34-41-dFc IgGB	**6.16E-09**	4.77E-11	**2.35E05**	3.63E00	**4.06E02**	2.41E-01	**1.45E-03**	9.09E-03	**1.09E-05**	5.65E-04	0.05	0.99
34-41-dFc IgGB	**5.55E-09**	4.10E-11	**2.42E05**	3.80E00	**4.95E02**	8.50E-02	**1.34E-03**	1.31E-02	**9.53E-06**	3.20E-04	0.06	0.99
34-41-dFc IgGB	**1.32E-09**	2.03E-11	**2.95E05**	5.40E-02	**1.39E03**	1.43E-03	**3.89E-04**	1.25E-03	**5.72E-06**	2.07E-05	0.07	0.99
34-41-hFc IgG1	**1.15E-09**	1.13E-11	**2.91E05**	1.83E-01	**9.22E02**	3.35E-03	**3.35E-04**	5.11E-03	**3.11E-06**	5.33E-05	0.07	0.99
34-41-hFc IgG1	**4.39E-09**	3.39E-11	**3.17E05**	3.89E00	**9.92E02**	1.43E-01	**1.39E-03**	2.72E-02	**9.82E-06**	1.02E-03	0.13	0.99
34-41-hFc IgG1	**5.68E-09**	4.23E-11	**2.81E05**	3.50E00	**5.95E02**	1.48E-01	**1.59E-03**	1.21E-02	**1.14E-05**	4.97E-04	0.07	0.99
34-dFc IgGA	**7.39E-10**	1.58E-11	**1.30E06**	6.78E-01	**5.30E03**	2.85E-02	**9.60E-04**	1.85E-03	**2.01E-05**	2.88E-05	0.01	0.99
34-dFc IgGA	**4.16E-09**	1.67E-10	**2.12E06**	1.97E01	**3.20E04**	1.72E-01	**8.81E-03**	1.94E-02	**3.28E-04**	4.44E-04	0.19	0.96
34-dFc IgGA	**2.51E-09**	3.26E-11	**1.05E06**	1.80E01	**4.75E03**	8.58E-05	**2.63E-03**	1.41E-02	**3.21E-05**	1.52E-04	0.04	0.99
34-dFc IgGB	**4.38E-09**	5.61E-11	**7.54E05**	1.28E01	**3.48E03**	4.50E-01	**3.30E-03**	2.24E-02	**3.95E-05**	8.25E-04	0.04	0.99
34-dFc IgGB	**4.51E-09**	6.59E-11	**5.65E05**	1.63E01	**4.06E03**	5.69E-01	**2.55E-03**	2.25E-02	**3.24E-05**	8.17E-04	0.04	0.99
34-dFc IgGB	**5.68E-09**	8.34E-11	**6.13E05**	1.17E01	**2.53E03**	1.51E00	**3.48E-03**	1.29E-02	**4.91E-05**	1.59E-03	0.03	0.99
34-hFc IgG1	**2.57E-09**	4.14E-11	**7.41E05**	1.24E01	**5.94E03**	4.17E00	**1.90E-03**	4.36E-02	**2.66E-05**	1.40E-02	0.08	0.99
34-hFc IgG1	**2.32E-09**	3.15E-11	**6.40E05**	2.01E01	**4.95E03**	1.22E-01	**1.49E-03**	4.66E-02	**1.66E-05**	1.13E-03	0.05	0.99
34-hFc IgG1	**4.70E-09**	9.19E-11	**9.34E05**	9.09E00	**5.16E03**	7.59E-01	**4.40E-03**	1.23E-02	**8.24E-05**	1.00E-03	0.10	0.99
41-34	**3.24E-08**	9.99E-10	**3.22E05**	1.01E01	**1.71E03**	4.17E00	**1.04E-02**	2.18E-02	**3.17E-04**	8.76E-03	0.04	0.99
41-34	**1.85E-08**	4.62E-10	**5.31E05**	9.48E00	**3.46E03**	4.05E00	**9.83E-03**	4.467E-02	**2.37E-04**	1.87E-02	0.07	0.99
41-34	**4.28E-09**	6.89E-11	**3.12E05**	4.95E-02	**1.89E03**	2.29E-03	**1.34E-03**	<1,0E-07	**1.99E-05**	<1,0E-07	0.06	0.99
41-34-dFc IgGA	**1.15E-09**	2.52E-11	**4.22E05**	1.04E-01	**2.99E03**	4.15E-03	**4.87E-04**	2.08E-03	**1.00E-05**	4.33E-05	0.21	0.99
41-34-dFc IgGA	**8.33E-09**	1.04E-10	**2.583E05**	3.197E00	**7.56E02**	8.03E-01	**2.15E-03**	1.11E-02	**2.62E-05**	2.63E-03	0.16	0.99

41-34-dFc IgGA	**4.43E-09**	6.14E-11	**3.22E05**	4.40E00	**1.46E03**	2.02E-01	**1.42E-03**	1.52E-02	**1.86E-05**	7.77E-04	0.31	0.99
41-34-dFc IgGB	**3.95E-09**	3.49E-11	**3.28E05**	2.66E00	**5.67E02**	3.15E-01	**1.30E-03**	5.48E-03	**1.12E-05**	5.57E-04	0.05	0.99
41-34-dFc IgGB	**4.52E-09**	2.03E-11	**2.92E05**	6.40E00	**3.25E02**	9.38E-04	**1.32E-03**	1.00E-02	**5.72E-06**	4.77E-05	0.06	0.99
41-34-dFc IgGB	**4.47E-09**	4.05E-11	**3.12E05**	6.16E00	**5.72E02**	6.97E-03	**1.40E-03**	5.57E-03	**1.24E-05**	2.91E-05	0.06	0.99
41-34-hFc IgG1	**2.34E-09**	2.02E-11	**4.04E05**	2.09E00	**1.01E03**	2.27E-01	**9.32E-04**	6.99E-03	**7.84E-06**	6.24E-04	0.09	0.99
41-34-hFc IgG1	**3.66E-09**	3.06E-11	**3.38E05**	6.24E00	**9.45E02**	2.59E-02	**1.24E-03**	1.44E-02	**9.74E-06**	1.99E-04	0.07	0.99
41-34-hFc IgG1	**2.81E-09**	1.87E-11	**3.79E05**	6.15E00	**5.34E02**	3.03E-02	**1.07E-03**	9.80E-03	**6.92E-06**	7.14E-05	0.04	0.99
41-dFc IgGA	**8.81E-09**	8.16E-10	**3.25E04**	9.14E-03	**1.23E03**	2.12E-03	**2.87E-04**	<1,0E-07	**2.41E-05**	<1,0E-07	0.11	0.99
41-dFc IgGA	**1.70E-09**	9.11E-11	**5.83E04**	3.30E-08	**3.67E02**	1.76E01	**9.92E-05**	2.78E00	**5.28E-06**	1.48E09	0.01	0.99
41-dFc IgGA	**7.03E-09**	2.48E-10	**4.57E04**	3.78E-03	**8.21E02**	8.39E-04	**3.21E-04**	<1,0E-07	**9.77E-06**	<1,0E-07	0.09	0.99
41-dFc IgGB	**5.84E-09**	1.49E-10	**2.48E05**	1.41E00	**1.54E03**	3.05E-01	**1.45E-03**	6.41E-03	**3.58E-05**	1.10E-03	0.24	0.99
41-dFc IgGB	**4.57E-09**	1.11E-10	**2.08E05**	1.30E00	**7.18E02**	1.92E-01	**9.52E-04**	2.96E-03	**2.29E-05**	2.89E-04	0.06	0.99
41-dFc IgGB	**2.57E-09**	7.78E-11	**2.10E05**	6.44E-02	**1.87E03**	3.40E-03	**5.40E-04**	1.13E-03	**1.56E-05**	3.96E-05	0.16	0.9
41-hFc IgG1	**1.40E-09**	2.54E-11	**7.50E05**	1.17E00	**5.29E03**	8.92E-02	**1.05E-03**	6.63E-03	**1.76E-05**	3.12E-04	0.34	0.99
41-hFc IgG1	**1.18E-09**	4.40E-11	**4.26E05**	7.77E-02	**7.15E03**	5.50E-03	**5.01E-04**	2.47E-03	**1.68E-05**	1.43E-04	0.36	0.99
41-hFc IgG1	**2.87E-09**	7.02E-11	**1.95E05**	6.05E-02	**1.35E03**	2.58E-03	**5.60E-04**	1.10E-03	**1.31E-05**	3.28E-05	0.11	0.99

Table S3: Characteristics of healthy PBMC dog donors

#	SEX	Age (years)	Breed	ASSAY
**0**	Female	5	Maltese	ADCC
**1**	Male	2	Beagle	cIFNγ production
**2**	Female	2	Beagle	cIFNγ production
**3**	Male	2	Beagle	cIFNγ production
**4**	Male	2	Beagle	cIFNγ production
**5**	Female	2	Beagle	cIFNγ production
**6**	Female	2	Beagle	cIFNγ production

SUPPLEMENTAL FIGURES & FIGURES LEGENDS

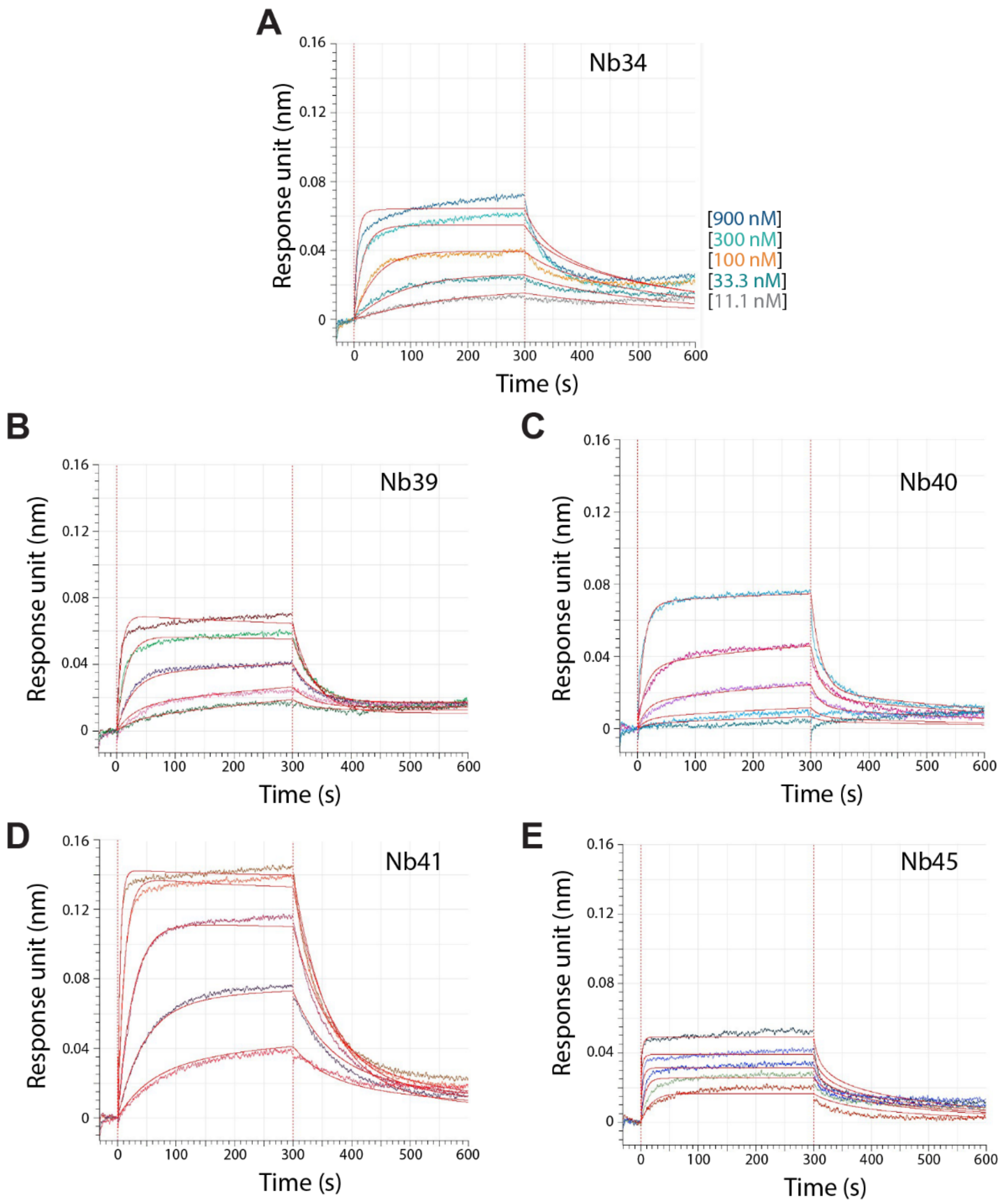

Figure S1: Binding of the anti-cPD-L1 nanobodies to recombinant cPD-L1 ectodomain

(**A-E**) Binding activity of the indicated Nbs against the immobilized cPD-L1 ectodomain were analyzed by BLI. Raw data are color-coded according to the assessed Nb-concentration. Fitted curves are plotted as red lines employing the Octet analysis studio software®. Vertical dotted red lines correspond to either the transition between the base line and the association phase (left) or the transition between the association and dissociation phases (right). Data were collected from *n* = 3 independent experiments using the Octet®R2 device. One representative experiment is displayed.

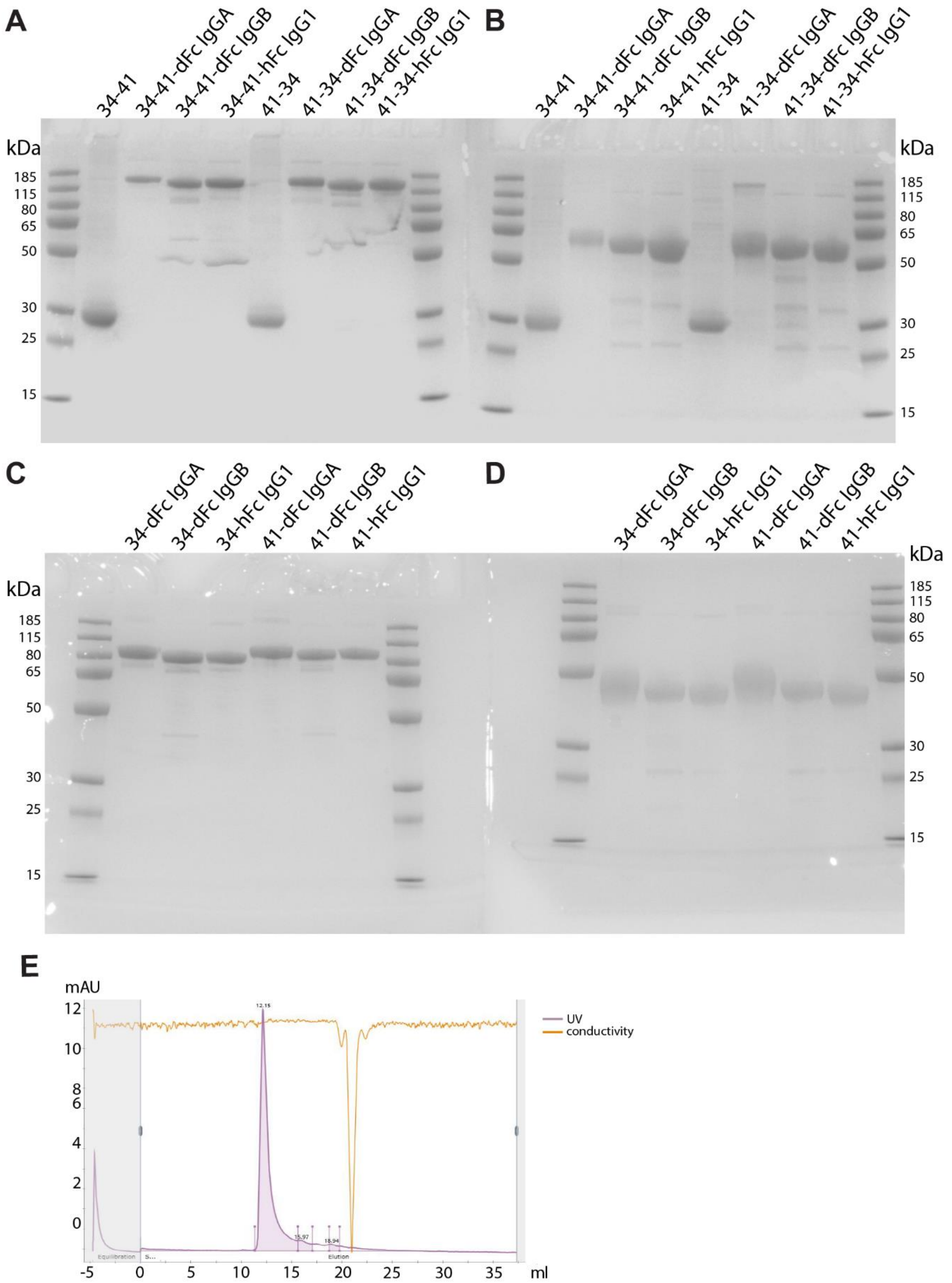

Figure S2: Biochemical characterization of Nb constructs.

Nb constructs were produced in mammalian cells and their molecular weight was confirmed by SDS-PAGE analyses ran under reducing (**B and D**) and nonreducing (**A and C**) conditions. (**E**) The purity of the Nb constructs was assessed by Size Exclusion Chromatography (SEC) using a

Superdex 200 increase 10/300 GL (Cytivia). One representative experiment is displayed with Nb 34-41-dFc IgGB.

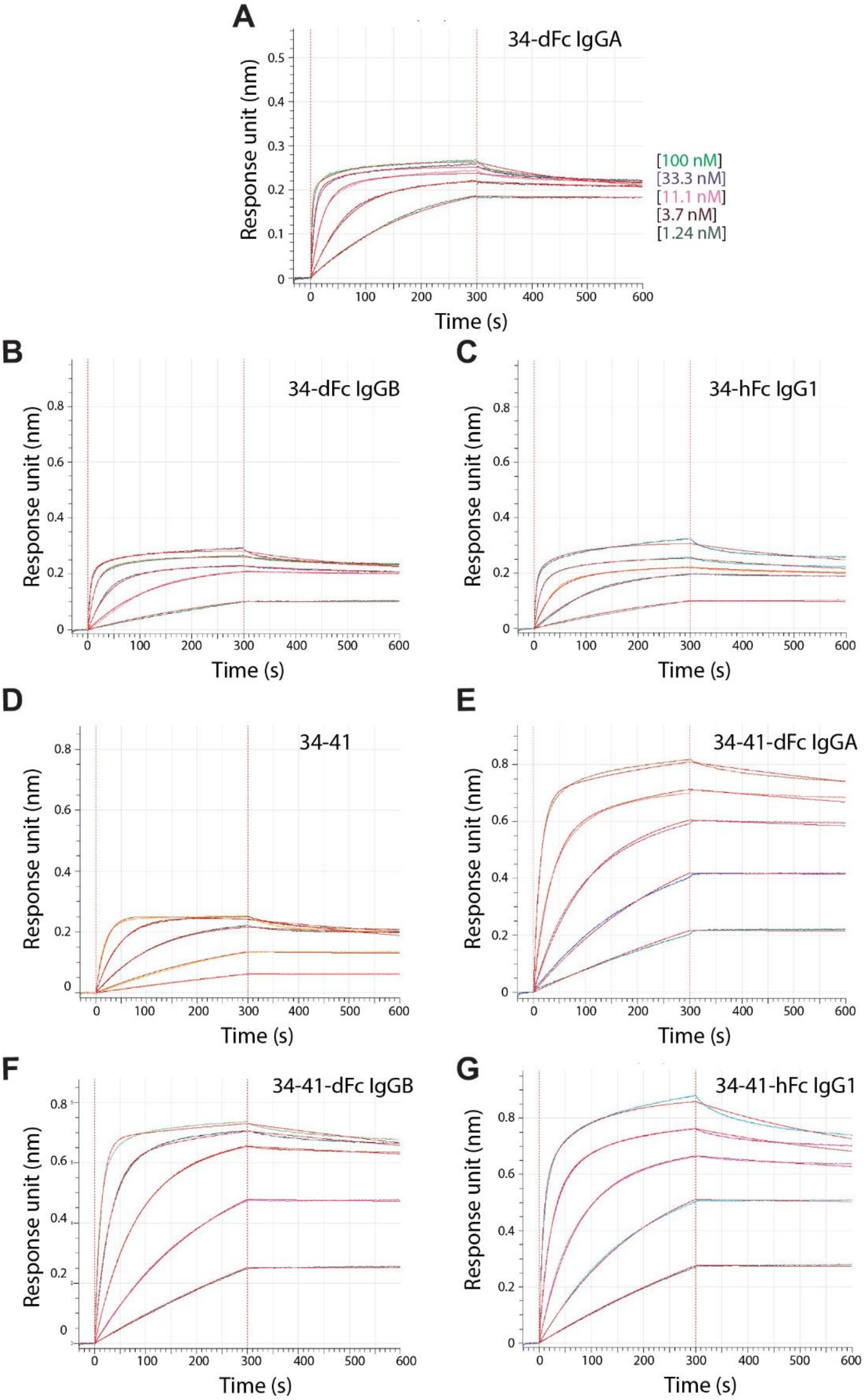

Figure S3: Binding of the Nb34-derived constructs to recombinant cPD-L1 ectodomain.

(**A-G**) Binding activity of the indicated Nbs against the immobilized cPD-L1 ectodomain were analyzed by BLI. Raw data are color-coded according to the assessed Nb-concentration (indicated on the right of panel (A)). Fitted curves are plotted as red lines employing the Octet

analysis studio software®. Vertical dotted red lines correspond to either the transition between the base line and the association phase (left) or the transition between the association and dissociation phases (right). Data were collected from *n* = 3 independent experiments using the Octet®R2 device. One representative experiment is displayed.

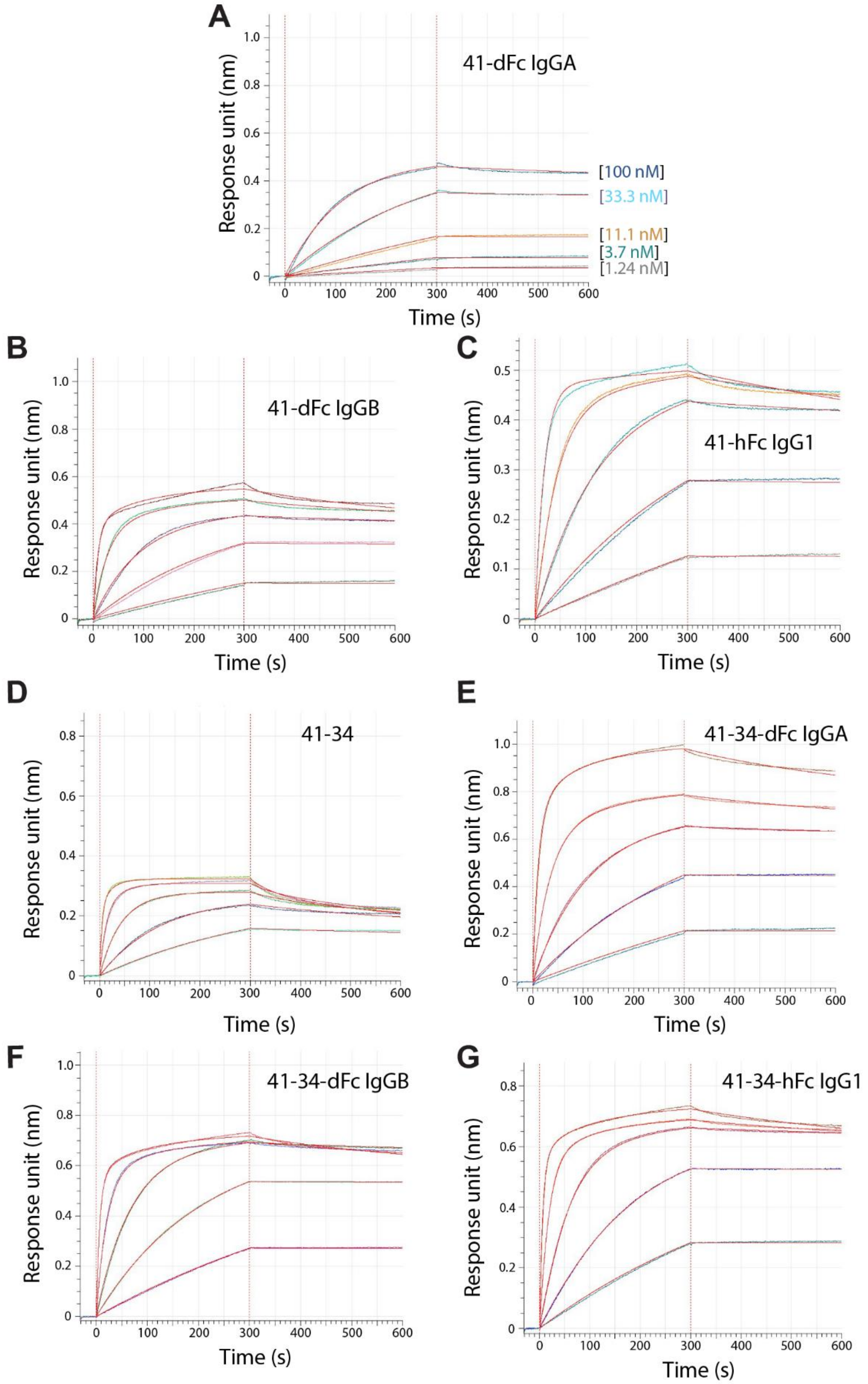

Figure S4: Binding of the Nb41-derived constructs to recombinant cPD-L1 ectodomain.

(**A-G**) Binding activity of the indicated Nbs against the immobilized cPD-L1 ectodomain were analyzed by BLI. Raw data are color-coded according to the assessed Nb-concentration (indicated on the right of panel (A)). Fitted curves are plotted as red lines employing the Octet analysis studio software®. Vertical dotted red lines correspond to either the transition between the base line and the association phase (left) or the transition between the association and dissociation phases (right). Data were collected from *n* = 3 independent experiments using the Octet®R2 device. One representative experiment is displayed.

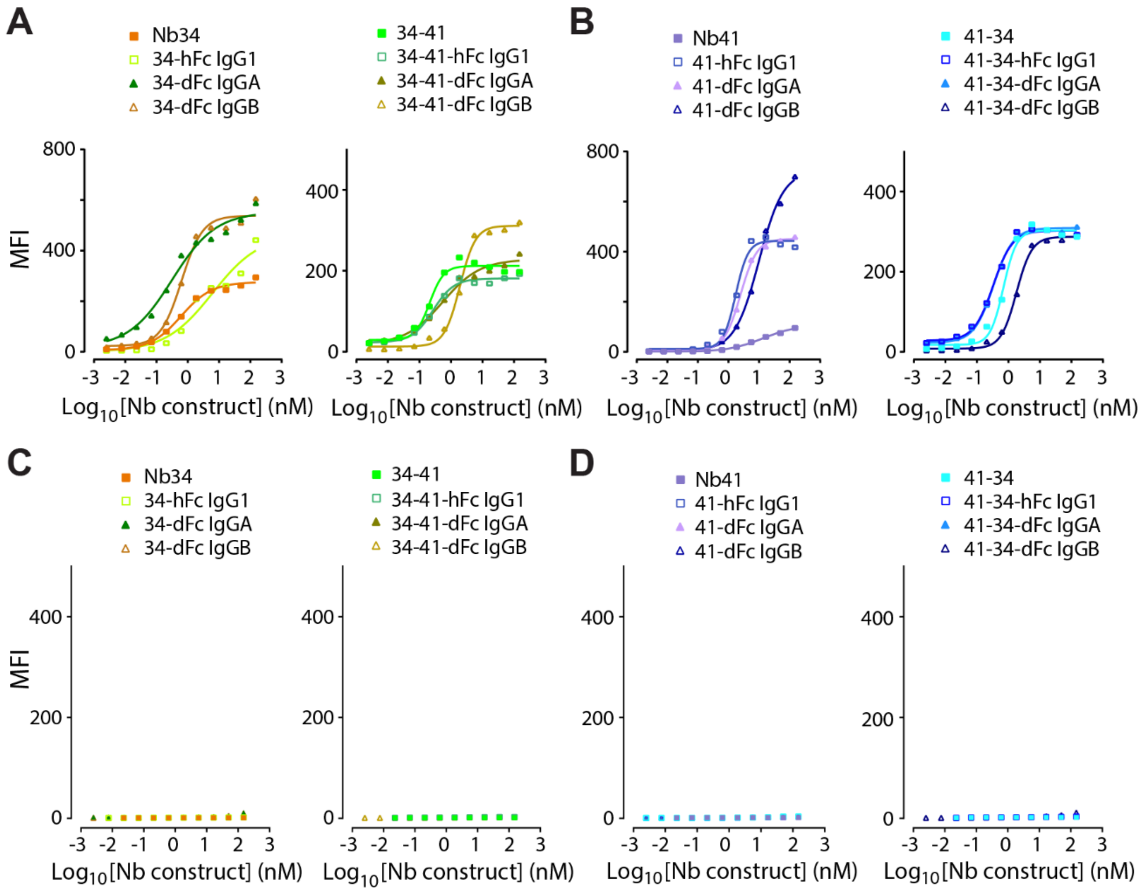

Figure S5: Binding of the Nbs and derivatives constructs to membrane-anchored cPD-L1.

Assessment of the binding efficiency of the Nbs and derivative constructs on cPD-L1-expressing HeLa (**A and B**) and (cPD-L1-untransduced) G06A (**C and D**) cells by flow cytometry. Binding was detected with a mouse anti-HIS followed by an Alexa eFluor647-conjugated goat anti-mouse IgG. Binding assessment was obtained from *n* = 3 independent experiments. One representative experiment is shown. MFI data were extracted from single cells gating using Flowlogic software™ and curves were plotted with GraphPad Prism v.10.

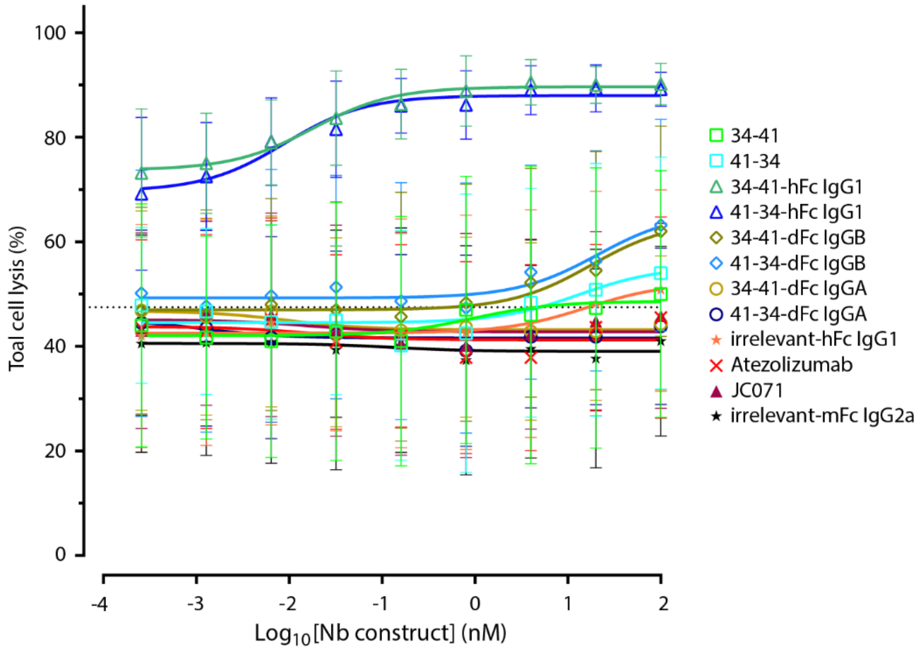

Figure S6: Assessment of the ADCC-inducing ability of the Nb constructs.

ADCC-triggering by the nanobody constructs was determined by employing canine PD-L1-expressing HeLa human cells co-cultured with purified human NK cells in the presence of Fc-carrying (or not) anti-canine PD-L1 Nb constructs. Cell lysis, induced by Fc-dependent activation of CD16-expressing NK cells, was recorded after 16h of incubation using Cell titer glo kit (Promega). Data are mean ± SD calculated from n = 3 biological replicate (donor) performed in technical duplicates.
