# Supplementary material for: Nanobody-based canine PD-L1-targeting immune checkpoint inhibitors for cancer therapy in dogs
Source: Mol Ther Oncol. 2025 Sep 8;33(3):201036. doi: 10.1016/j.omton.2025.201036 (PMC12587322; doi:10.1016/j.omton.2025.201036)
Supplement: Document S2. Article plus supplemental information [file mmc2.pdf]

Molecular Therapy
Oncology
Original Article

Nanobody-based canine PD-L1-targeting immune checkpoint inhibitors for cancer therapy in dogs

Morgane Di Palma Subran,[1,6] Marianne Wyss,[2] Betül Taskoparan,[3] Mathischan Maheswaran,[3] Johannes vom Berg,[3] Philippe Plattet,[2,4,5] and Patrick Chames[1,5]

[1]Aix Marseille Univ, CNRS, INSERM, Institut Paoli-Calmettes, CRCM, Marseille, France; [2]Division of Neurological Sciences, Vetsuisse Faculty, University of Bern, Bern, Switzerland; [3]Institute of Laboratory Animal Science, University of Zurich, Schlieren, Switzerland; [4]Multidisciplinary Center for Infectious Diseases (MCID), University of Bern, Bern, Switzerland

Although the demand for novel immunotherapies to treat companion dogs with spontaneously developing cancer is increasing in high-income countries, most options remain inaccessible. Dogs host a complete and functional immune system reacting to the presence of their tumor. As for humans, many canine neoplasms were shown to overexpress programmed death-ligand 1 (PD-L1), an immune checkpoint inhibitor (ICI) known to downregulate cytotoxic T cell activity upon interaction with its ligand PD-1. In this study, we used alpaca-derived single domain antibodies (sdAbs), also known as nanobodies (Nbs), to develop new ICI targeting canine PD-L1. We selected several clones binding to both recombinant soluble and cell membrane-anchored cPD-L1 forms. Next, their cPD-L1-binding affinities, cPD-1/cPD-L1-blocking abilities and epitope relationships were determined. Most effective Nbs binding to non-overlapping epitopes were combined as biparatopic Fc fusions to provide additional functionalities and improve their efficacy. Remarkably, all engineered Nb constructs efficiently interfered with the cPD-1/cPD-L1-induced signaling pathway, with some multivalent molecules displaying inhibitory concentrations reaching low picomolar range. Moreover, Fc-competent Nb constructs were also shown to induce tumor cell death by antibody-dependent cell-mediated cytotoxicity using human or canine models. Finally, using donor canine peripheral blood mononuclear cells (PBMCs), best candidates were favorably compared with atezolizumab in a Staphylococcal enterotoxin B (SEB)-based interferon-γ (IFNγ) secretion assay.

INTRODUCTION

The development of immune checkpoint blockade (ICB) has drastically changed cancer treatment approaches over the last decade. Immune checkpoints are physiologically used by cells to maintain self-tolerance and prevent autoimmunity.[1,2] Cells expressing several ligands at their surface engage those inhibitory receptors present on immune cells. One of the most characterized immune checkpoints is the PD-1/PD-L1 axis. The receptor, programmed death protein 1 (PD-1, also known as CD279) is expressed by activated T cells in peripheral tissues. It binds to two different ligands, PD-L1 and PD-L2, commonly expressed on antigen-presenting cells, such as dendritic cells or macrophages to regulate T cell activity.[3] PD-L1 can also be found on non-lymphoid tissues, non-hematopoietic cells such as epithelial cells, hepatocytes, and vascular endothelial cells.[2]

However, during tumorigenesis, numerous tumors express PD-L1 to downregulate the immune response and create an immunosuppressive microenvironment.[4] The PD-1/PD-L1 axis contributes to inhibiting T cell proliferation, cytokine release, and cytotoxicity, leading to the exhaustion of tumor-specific T cells.[5] The blockade of this pathway using anti-PD-1 or anti-PD-L1 antibodies has led to durable responses in cancer treatment.[6] Seven anti PD-1 antibodies (nivolumab, pembrolizumab, cemiplimab, retifanlimab, tislelizumab, and toripalimab) and three anti PD-L1 antibodies (atezolizumab, avelumab, and durvalumab) are now approved by the European Medicines Agency and the United States Food and Drug Administration (FDA) for various human cancers including melanoma, non-small cell lung carcinoma, and renal cancer.[7] Nevertheless, a majority of patients develops intrinsic and acquired resistance[8] that is not easily recapitulated by murine models currently used in preclinical studies. Indeed, artificially induced tumors do not faithfully mimic the high cancer heterogeneity or the complex mutational burden of human cancers, continuously shaped by the cancer-host immunoediting process.[9] Moreover, the microbiome of laboratory mice, known to strongly affect immunotherapies,[10] deviates substantially. In contrast, companion dogs are exposed to the same environment as their owners. Additionally, some studies suggest that the

Received 9 January 2025; accepted 14 August 2025;
https://doi.org/10.1016/j.omton.2025.201036.

[5]These authors contributed equally

[6]Present address: Division of Neurological Sciences, Vetsuisse Faculty, University of Bern, Bern, Switzerland

Correspondence: Philippe Plattet, Division of Neurological Sciences, Vetsuisse Faculty, University of Bern, Bern, Switzerland.
E-mail: philippe.plattet@unibe.ch

© 2025 The Author(s).
Published by Elsevier Inc. on behalf of The American Society of Gene and Cell Therapy.
This is an open access article under the CC BY license (http://creativecommons.org/licenses/by/4.0/).

microbiome is relatively shared across species (23%) when living in close proximity.[11,12] According to this notion, canine spontaneous cancers may provide more accurate and predictive models.[9,13] Indeed, despite bearing a complete and functional immune system, many dogs develop PD-L1 overexpressing tumors, including oral melanoma, lymphoma, melanoma, mast cell tumors, fibrosarcoma, hepatocellular carcinoma, or renal cell carcinoma.[14–18] In some studies, the blockade of PD-1/PD-L1 pathway was shown to significantly enhance interferon-γ (IFNγ) production and T cell proliferation.[18,19] Of note, none of these tested antibodies were specifically developed for dogs. There is currently no immunotherapy based on the cPD-L1 approach approved in veterinary medicine. However, several teams recently developed anti-cPD-L1 antibodies using different approaches. Sirivisoot et al. developed a mouse anti-canine PD-L1 as a prognostic marker for canine tumor.[20] Other studies have demonstrated the efficiency of an anti-cPD-L1 as a treatment.[21–25] For example, a canine chimeric anti-PD-L1 monoclonal antibody has been developed to treat oral malignant melanoma in dog.[21] The result of this study and the work of Choi et al., who developed several monoclonal mouse antibodies against cPD-L1,[23] showed efficient inhibition of the binding of cPD-1 to cPD-L1, an enhanced IFNγ production by dog peripheral blood mononuclear cells (PBMCs), and increased production of T lymphocytes. Yoshimoto et al. developed a fully canine high affinity anti cPD-1 effectively inhibiting cPD-1/cPD-L1 interaction.[26] Their study demonstrates the benefits of developing immunotherapies in dogs that can not only improve veterinary medicine but also may be valuable in informing future human clinical trial designs. Thus, developing canine antibodies with favorable properties that can be economically produced on a clinical scale could be of major interest.

In this work, we have identified and characterized a panel of potent anti cPD-L1 nanobodies (Nbs), some of them being able to block the cPD-1/cPD-L1 interaction. We then took benefit of their smaller size, favorable physico-chemical properties and modularity to engineer a variety of multivalent formats, including biparatopic tandems as well as various biparatopic Fc fusions. We finally compared their ability to block the cPD-1/cPD-L1 inhibitory axis in reporter cells or primary canine cells, as well as the potential benefit of using a competent canine Fc to engage immune cells *in vitro*.

RESULTS

Discovery of potent anti-canine (c)PD-L1 binders

Nbs were obtained from an alpaca immunized with a commercially available soluble canine PD-L1 ectodomain, followed by several rounds of panning by phage display. This resulted in the isolation of five clones binding to both soluble PD-L1 recombinant protein as well as to the full length, membrane-bound form of the protein, as determined by bio-layer interferometry (BLI) (Figure S1) and flow cytometry (Figure 1A) analyses. While the binding affinities of the Nbs to the recombinant protein ranged from 2 μM to 5 nM, their apparent affinities on cPD-L1-expressing cells ranged from 150 to 4 nM (Table 1). The ranking of the Nbs for both methods was similar, with Nb34 exhibiting the strongest binding efficacy. We next investigated the ability of each Nb to block the canine PD-1/PD-L1 interaction by competition ELISA. Strikingly, all Nbs except Nb41 successfully blocked the cPD-1/cPD-L1 interaction, with IC_{50} values ranging from 340 to 53 nM (Figure 1B). Since canine PD-L1 exhibits about 80% amino acid similarity with hPD-L1, we also determined whether the Nbs could cross-react with the human PD-L1 molecule, using flow cytometry and hPD-L1-transduced M113 cells. None of the Nbs cross-reacted with hPD-L1 (Figure 1C).

To determine whether the various identified Nbs shared their epitopes on cPD-L1, we conducted an epitope binning experiment by employing a BLI binding assay. Upon immobilization of soluble cPD-L1 on the sensor, a first Nb was injected at equilibrium phase concentration. Next, we monitored the binding activity of the second Nb, added in the presence of the first one. While no variation in the signals indicates a putative competition between the two Nbs for the same epitope, an increased signal suggests a simultaneous binding of both Nbs at two non-overlapping epitopes on cPD-L1. We also investigated whether the discovered Nbs additionally competed with the commercially available anti-canine PD-L1 monoclonal antibody (mAb) JC071, previously reported to block the cPD-1/cPD-L1 interaction.[27] Interestingly, among the four Nbs that blocked the cPD-1/cPD-L1 interaction, Nb34 and Nb39 appeared to bind cPD-L1 in close proximity (Figure 1D) but only hindered the binding of Nb40 and Nb45 to some extent (limited signal inhibition), suggesting a partial overlapping with their epitope. However, Nb40 and Nb45 likely bind to non-overlapping epitopes as no signal inhibition was measured between them (Figure 1D). A schematic summary of the epitope mapping is presented in Figure 1E. Surprisingly, JC071 solely competed with Nb41, i.e., the only Nb that did not block the cPD-1/cPD-L1 interaction in our competition ELISA (Figure 1B). Because mAbs consist of much larger molecules as compared to monovalent Nbs, the reported cPD-1/cPD-L1 blocking ability of JC071 may thus rely on steric hindrances.

Design of Nb-based multivalent constructs

To potentially improve the likelihood of generating molecules matching clinical requirements, we designed various multivalent constructs. First, we sought to increase the binding affinity to cPD-L1 by engineering biparatopic constructs. To this aim, we selected a Nb pair that (1) bound effectively to cPD-L1 and (2) interacted at two non-overlapping epitopes (i.e., Nb34 and Nb41). The two Nbs were fused via a flexible linker, in both orientations (34-41 or 41-34; Figure 2A). We also fused Nb34 and Nb41 to Fc portions to enhance the overall valency of the molecule, prolong their half-life in the bloodstream, and to investigate in future experiments whether Fc-related effector functions may be beneficial *in vivo*. We selected three Fc portions: two reported to trigger the recruitment of effector functions (human IgG1 and dog IgGB), as well as one defective (dog IgGA).[28] Fc portions were fused to the C-terminus of the monovalent and biparatopic Nbs (Figure 2A). All Nb constructs were produced in mammalian cells and purified using protein A (Fc-carrying constructs) or immobilized metal affinity chromatography (IMAC)

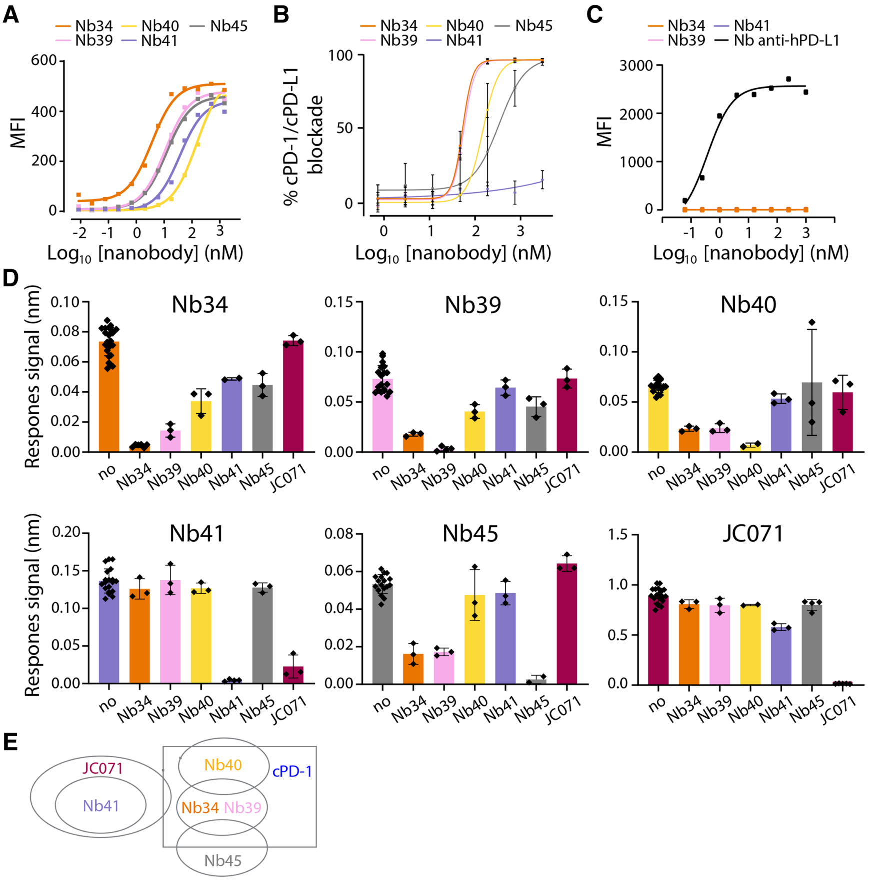

Figure 1. Characterization of anti-cPD-L1 nanobodies

(A) Binding determination of the indicated Nbs on cPD-L1-expressing HEK293T cells by flow cytometry. Binding was detected with a mouse anti-HIS followed by an Alexa Fluor 647-conjugated goat anti-mouse IgG. Binding assessment was obtained from n = 3 independent experiments. Raw data of one representative experiment is shown. MFI data were extracted from single cells gating using Flowlogic software and curves were plotted with GraphPad Prism v.10. (B) Investigation of the ability of the various Nbs to block cPD-1/cPD-L1 interaction by ELISA. cPD-1-Fc was immobilized on plate and followed by the addition of indicated Nbs. Plates were exposed to biotinylated cPD-L1-Fc, washed and streptavidin-HRP was used for detection. Data are mean ± standard deviation (SD) calculated from n = 3 independent experiments. (C) Binding determination of the indicated Nbs on human PD-L1-expressing M113 cells by flow cytometry. Binding was detected with a mouse anti-HIS followed by an Alexa Fluor 647-conjugated goat anti-mouse IgG. Binding assessment was obtained from n = 1 experiment. MFI data were extracted from single cells gating using Flowlogic software and curves were plotted with GraphPad Prism v.10. (D) Epitope binning experiments investigated by BLI. Upon immobilization of biotinylated cPD-L1-Fc on streptavidin probe, a first Nb was incubated (100 or 300 nM). Subsequently a second Nb was supplemented (100 or 300 nM) in the presence of the first Nb. Data are mean ± SD calculated from n = 3 independent experiments. Values were recorded with the OctetR2 device and data analyses performed with the Octet analysis studio software. Histograms were plotted using GraphPad Prism v.10. (E) Schematic representation summarizing the epitope binning experiments.

(Fc-less constructs) columns (Table S1). Protein quality control was determined by SDS-PAGE (Figures S2A–S2D) and size exclusion chromatography (Figure S2E).

Determination of the binding efficacy of the various Nb constructs was investigated against soluble recombinant cPD-L1 using BLI, and against full length membrane-anchored cPD-L1 molecules expressed by cPD-L1-transduced human or canine cells (i.e., HeLa and G06A, respectively) using flow cytometry. Collectively, all engineered molecules potently interacted with cPD-L1, although no major binding enhancement was observed compared to the monovalent Nbs (Figures 2B and 2C; Table 2). However, most constructs exhibited >5-fold affinity increase on the membrane-anchored cPD-L1 protein (Table 2). Overall, binding efficiencies typically reached values in the single digit nM range, except for the biparatopic constructs, which exhibited dissociation constant (K_D) values of about 18 nM when recorded by BLI (Figures S3 and S4; Table 2; Table S2). Similar results were obtained when assessed using different cell lines, including cPD-L1 transfected HeLa cells (Figures S5A and S5B). Of note, no signal was detected in non-transduced G06A cells (Figures S5C and S5D), thereby indicating that the canine brain cells did not efficiently express cPD-L1 at their surface.

Nb-Fc fusions interfere with cPD-1/cPD-L1-induced signaling pathway

We investigated the ability of our panel of constructs to interfere with the signaling pathway induced upon the interaction of cPD-L1 and cPD-1 on cells. To this aim, we employed a newly engineered cell-based reporter assay (Promega) that is equivalent to a previously reported system relying on the human PD-1 and PD-L1 molecules. The assay relies on mixing two cell populations, which results in the inhibition of the T cell receptor (TCR) signaling and NFAT-mediated luciferase activity upon productive interaction between PD-1 and PD-L1. The assay enables a quantitative assessment of the PD-1/PD-L1 interaction blockade that disrupts the inhibitory signal and restores luciferase activity (Figure 3A).

Validating the data obtained in our blockade ELISA assay with monovalent Nbs (Figure 1B), all Nb constructs were able to increase luciferase activity, except for the non-blocking monovalent Nb41

Table 1. Binding affinities of the nanobodies

	Binding efficiency			
	Membrane-anchored protein[a]			
	HEK-cPD-L1 cells		Soluble protein[b]	
Binder	K_D app (nM)	SD (nM)	K_D (nM)	SD (nM)
34	4.3	1.6	4.47	0.08
39	7.7	2.6	8.84	0.12
40	134.80	20.00	1883.27	36.95
41	18.70	13.00	12.59	0.11
45	8.90	4.80	5.46	0.19

[a]Binding efficiency determined by flow cytometry.
[b]Binding efficiency determined by BLI.

(Figure 3B). However, the incorporation of Nb41 in our tandem constructs had a considerable impact, since they surpassed the blocking efficacy of Nb34 alone (Figure 3C). Interestingly, Nb41-Fc and mAb JC071 showed similar profiles, only leading to a slight enhancement of luciferase activity at high concentrations (Figures 3D and 3E), suggesting again a steric hindrance effect rather than a direct competition for the epitope targeted by cPD-1. Strikingly, the bivalent constructs were able to decrease the EC_{50} by about 100-fold as compared to monovalent Nbs, with values reaching the picomolar range (Figure 3F; Table 2). Furthermore, the tetravalent dFc molecules (Figures 3I and 3J) (but not the corresponding hFc constructs) were about 1,000-fold more efficient than the monovalent Nbs (Figure 3A) and 10-fold more efficient than the bivalent ones (Figures 3E–3G). Best tetravalent constructs were also about 100-fold more potent as compared to atezolizumab; an FDA-approved anti-human PD-L1 mAb (Figure 3D). Collectively, these findings highlight the successful engineering of multivalent Nb constructs that exhibited highly potent interference with the cPD-1/cPD-L1-mediated signaling pathway.

Nbs equipped with dog IgGB or human IgG1 Fc domains trigger ADCC

The hFc IgG1 and dFc IgGB domains were previously reported to productively recruit effector functions in a species-independent manner.[28] We thus investigated the ability of our anti-canine PD-L1 biparatopic constructs carrying a functional Fc domain (hIgG1 and dIgGB) to trigger antibody-dependent cell-mediated cytotoxicity (ADCC). To this aim, we co-cultured purified human natural killer (NK) cells with cPD-L1-expressing HeLa cells in the presence of a serial dilution of various Nb constructs. Sixteen hours post co-culture, human NK cells were washed and cell lysis was determined using the CellTiter-Glo kit (Promega) (Figure S6). As anticipated, biparatopic constructs carrying a functional human Fc domain (hFc IgG1) could trigger ADCC in a very efficient manner (low picomolar range). Interestingly, Nbs equipped with a functional canine Fc domain (dFc IgGB) could also induce ADCC to some extent, although in this case, cell lysis was recorded only at high drug concentrations (100 nM) (Figure S6). Those results were not unexpected considering the heterotypic nature of the system used (human NK cells and Nbs carrying a canine Fc domain).

We next determined the ability of the biparatopic constructs (equipped with hIgG1 and dIgGB Fc domains) to trigger ADCC

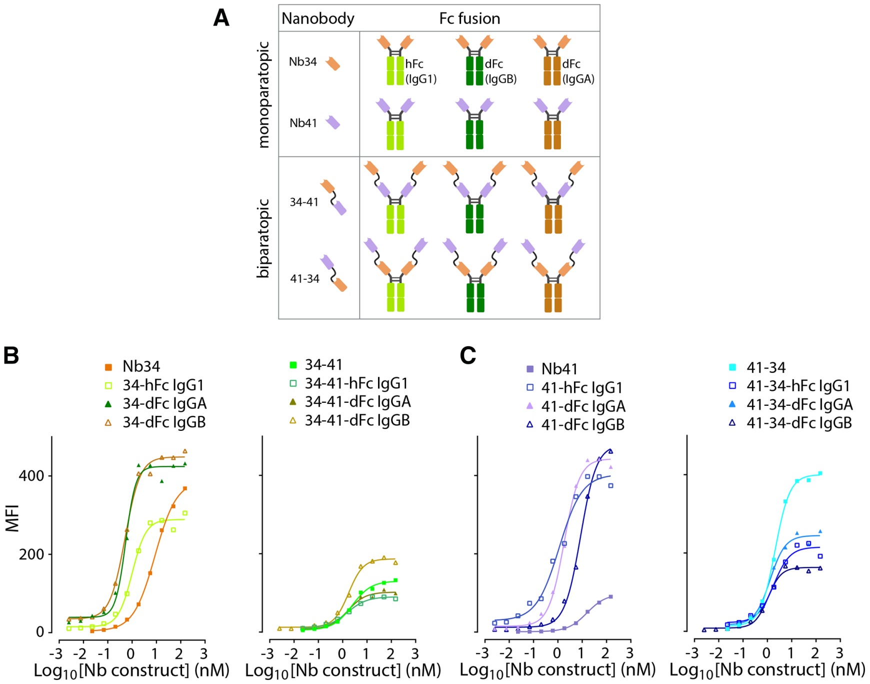

Figure 2. Binding of Nbs and derivative constructs to membrane-anchored cPD-L1
(A) Schematic representation of the diverse multivalent Nb constructs engineered in this study. (B and C) Assessment of the binding efficiency of the Nbs and derivative constructs on cPD-L1-expressing G06A canine cells by flow cytometry. Binding was detected with a mouse anti-HIS followed by an Alexa Fluor 647-conjugated goat anti-mouse IgG. Binding assessment was obtained from n = 3 independent experiments. One representative experiment is shown. MFI data were extracted from single cells gating using Flowlogic software and curves were plotted with GraphPad Prism v.10.

Table 2. Characterization of the nanobody constructs

	Binding efficiency							
	Membrane-anchored protein[a]							
	G06A-cPD-L1 cells		HeLa-cPD-L1 cells		Soluble protein[b]		cPD-1/cPD-L1 blockade	
Binder	K_D app (nM)	SD (nM)	K_D app (nM)2	SD (nM)	K_D (nM)	SD (nM)	EC50 (nM)	SD (nM) 2
34	4.17	3.84	3.30	2.52	4.47	0.08	67	47.26
34-41	1.77	0.70	0.30	0.17	17.5	0.43	0.52	0.48
34-41 dFc IgGA	3.07	2.72	0.87	0.90	4.60	0.06	0.04	0.02
34-41 dFc IgGB	1.50	0.26	0.43	0.21	4.30	0.04	0.04	0.02
34-41 hFc IgG1	1.10	0.40	0.23	0.12	3.70	0.03	0.90	0.07
34 dFc IgGA	1.00	0.95	0.90	1.39	2.50	0.07	0.43	0.08
34 dFc IgGB	4.10	3.25	1.60	1.84	4.80	0.07	0.43	0.26
34 hFc IgG1	1.83	1.62	5.10	6.93	3.20	0.05	0.71	0.17
41	10.27	6.90	29.63	17.52	12.59	0.11	N/A	N/A
41-34	1.80	0.26	0.63	0.06	18.40	0.51	0.42	0.25
41-34 dFc IgGA	2.47	2.45	0.77	0.90	4.60	0.06	0.05	0.02
41-34 dFc IgGB	0.77	0.40	0.20	0.10	4.30	0.03	0.03	0.01
41-34 hFc IgG1	1.03	0.58	0.23	0.06	2.90	0.02	0.35	0.10
41 dFc IgGA	9.73	3.83	14.00	4.00	5.80	0.38	N/A	N/A
41 dFc IgGB	2.80	2.18	1.00	0.69	4.30	0.11	N/A	N/A
41 hFc IgG1	1.93	1.71	0.80	0.78	1.80	0.05	N/A	N/A

N/A, not applicable.
[a]Binding efficiency determined by flow cytometry.
[b]Binding efficiency determined by BLI.

under more relevant conditions. We thus either co-cultured cPD-L1-expressing canine G06 cells with canine PBMCs (cPBMCs), or human cells expressing cPD-L1 (HeLa-cPD-L1) with human PBMCs (hPBMCs) (Figure 4A). Cells lysis was then monitored using real-time cell analyses (RTCA). This assay enables the real time follow-up of adherent target cell cytolysis in the presence of effector cell in suspension. Because atezolizumab is well known to be (1) impaired in Fc-dependent triggering of ADCC and (2) able to efficiently disrupt the PD-1/PD-L1 interaction of human and canine origins,[29] it was included as a negative control. Another control construct bearing the hFc IgG1 domain fused to an irrelevant Nb was also included.

As expected, when canine cells stably expressing cPD-L1 were mixed with canine PBMCs, cell lysis was exclusively recorded in the presence of Fc bearing antibodies exposing either the dFc IgGB or the hFc IgG1 domains (Figure 4B). Similar results were obtained when HeLa-cPD-L1 cells were mixed with hPBMCs, although a higher spontaneous cell lysis activity in the absence of antibody was recorded (Figure 4C). The displayed results correspond the time points exhibiting the highest difference between our Nb constructs and controls, i.e., 45 min or 7 h post-treatments in conditions employing the dog or human cells, respectively. This difference likely explains the undetectable spontaneous cell lysis in experimental conditions using canine cells, as compared to human cells (Figures 4B and 4C). Interestingly, we noticed a marginal induction of cell death mediated by atezolizumab in the presence of cPBMCs (Figure 4B). Although the N297A mutation of its Fc portion was design to reduce ADCC and effector functions in human settings, limited productive interactions between the engineered Fc domain and the FcγRs of canine origin could explain this result.[30,31]

Collectively, these data demonstrate that our Nb constructs harboring either the hFc IgG1 or dFc IgGB portions were able to significantly trigger ADCC. Our findings also confirm the notion that species-specificity is not required regarding the interaction of the hFc IgG1 or dFc IgGB domains with Fcy receptors of human or canine origins.

Nb-Fc fusions enhance IFNγ release from activated canine T cells

To further characterize the activity of our constructs with regards to T cell activation, we assessed whether the blockade of PD-L1 by our biparatopic Fc fusion Nbs or atezolizumab would result in an enhanced release of IFNγ by canine lymphocytes derived from healthy donors. The assay is based on the stimulation of canine PBMCs using Staphylococcal enterotoxin B (SEB), a toxin produced by the gram-positive bacteria *Staphylococcus aureus* and originally described for the validation of ICIs in human PBMCs.[32] Due to the simultaneous binding of SEB to (1) major histocompatibility complex class II molecules exposed by antigen-presenting cells (e.g., monocytes and B cells) and (2) TCR complexes, a substantial

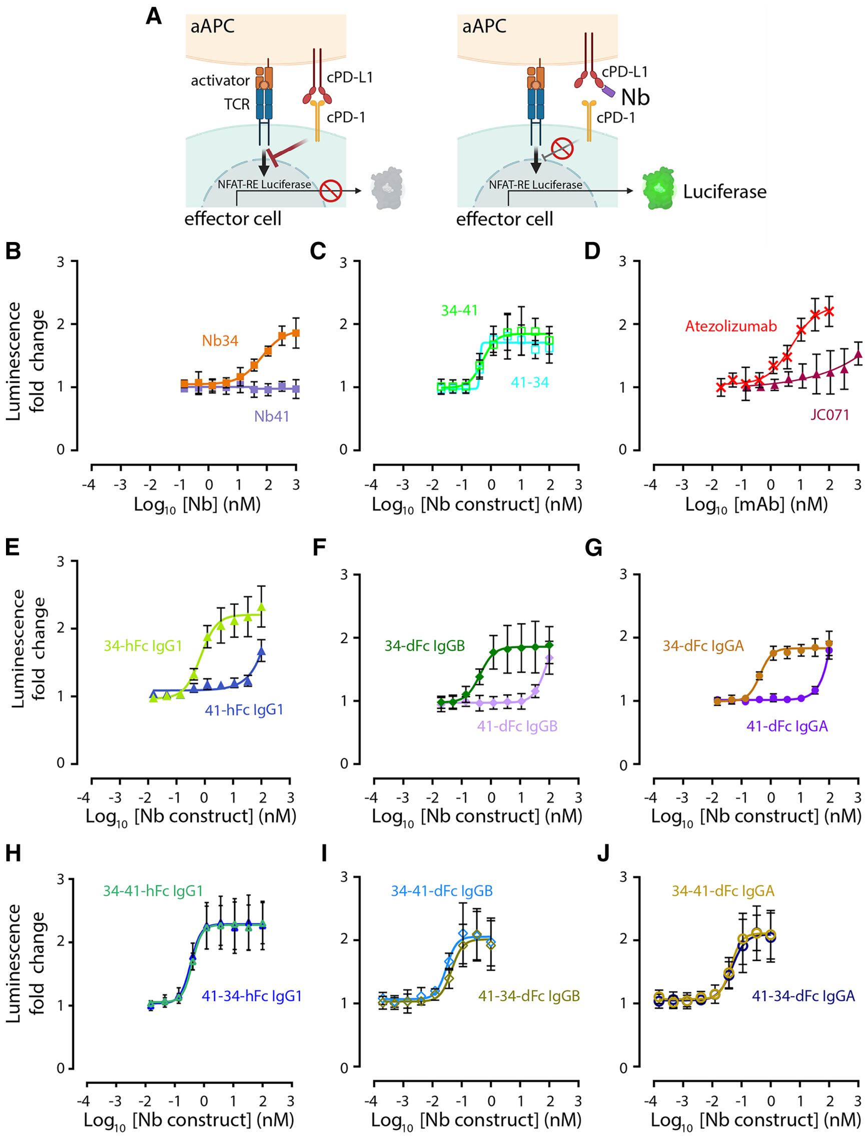

Figure 3. Determination of Nbs and derivative constructs to block the cPD-L1/cPD-1 interaction
(A) Cartoon illustrating the cell-based reporter assay used to evaluate the effect of the cPD-1/cPD-L1 blockade of Nb constructs (Promega). Upon mixing of cPD-1 positive effector cells with cPD-L1 expressing artificial antigen-presenting cells (aAPC cells), cells were treated with a potential inhibitor. Luminescence, driven by NFAT promotor was quantified upon addition of Bio-Glo reagent. (B–J) Depictions of the cPD-1/cPD-L1 blockade efficiency in the presence of increasing concentrations of the indicated Nb or derivative construct. Data are mean ± SD calculated from n = 3 independent experiments performed in technical triplicates. Curves were plotted using GraphPad Prism v.10.

number of T cells are being activated.[33] This results in the release of pro-inflammatory cytokines such as IFNγ by activated T cells, which is further modulated by the cPD-1/cPD-L1 axis[34] (Figures 5A and 5B). In this assay, atezolizumab modestly increased IFNγ production by cPBMCs in all donors (Figure 5C), compared to the control condition (Figure 5D). Notably, the two biparatopic constructs carrying the canine Fc-silent isotype appeared more active in most donors (Figure 5C), with the 34-41-dFc IgGA construct showing a statistically significant increase in IFNγ production (Figure 5D).

DISCUSSION

The overexpression of PD-L1 by cancer cells is one of the main strategies deployed by tumors to dampen the activity of tumor-specific T cells expressing the immune checkpoint PD-1. Cancer immunotherapy has made significant strides in the treatment of cancer, particularly using anti-PD-1/PD-L1 mAbs. However, the intrinsic and acquired resistance observed with these therapies in the clinical setting is currently not efficiently predicted by murine models. Tumors arising spontaneously in immune competent companion dogs could provide more predictive models. Moreover, cancer immunotherapy in veterinary medicine needs further development. Although newly developed monoclonal antibodies against canine PD-L1 demonstrated great potential as cancer therapeutics,[21–25] to date, none of these treatments are commercially available.

To fill this gap, we developed novel Nb-derived anti-PD-L1 constructs. Due to their small size and favorable physico-chemical properties, which allow them to be used in a variety of constructs, Nbs are considered attractive tools for the engineering of immunotherapeutics. In this study, we isolated five canine-specific anti-PD-L1 Nbs with a wide range of apparent affinity for cPD-L1. Noteworthily, four out of the five discovered Nbs could also efficiently block the cPD-1/cPD-L1 interaction. To potentially increase their functionality and reach, in the future, drug products with improved clinical profiles, multivalent constructs were also engineered. While biparatopic Nb-based constructs can simultaneously bind to two distinct epitopes of the same target, Fc fusions enable the generation of bivalent molecules. Regarding the design of biparatopic molecules, we selected Nb34, which exhibited best binding profiles to both the recombinant soluble and membrane-bound cPD-L1 proteins while efficiently interfering with the cPD-1/cPD-L1 interaction. On the other hand, and despite its inability to disrupt the engagement of cPD-L1 to cPD-1, Nb41 was nonetheless chosen because it interacts with cPD-L1 on a non-overlapping epitope. Although binding affinities to cPD-L1 of the resulting biparatopic molecules were not

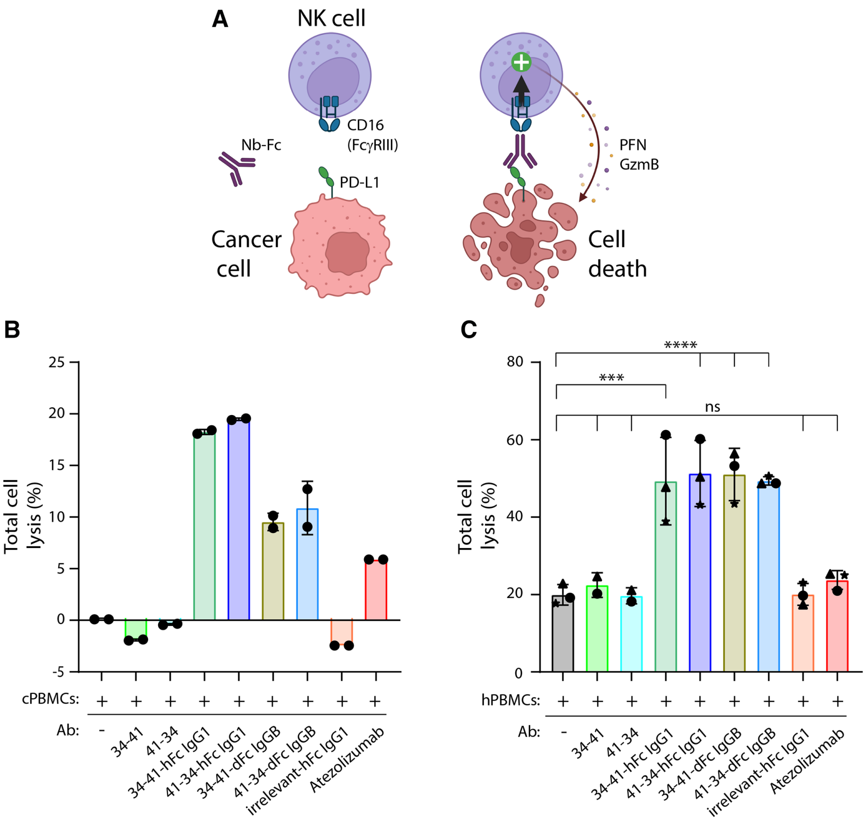

Figure 4. ADCC-triggering by Nb constructs

(A) Cartoon illustrating the cell-based assay employed to assess the induction of antibody-dependent cell-mediated cytotoxicity (ADCC) via the Fc portion of the diverse Nb constructs. Cancer cells were co-cultured with PBMCs in the presence of an Fc-carrying anti-PD-L1 construct. Target cell death, induced by Fc-dependent activation of CD16-expressing NK cells, was recorded by real-time cell analysis (RTCA, xCELLigence) measuring the variation of impedance correlated to the adherence of target cells. (B and C) (B) cPD-L1-expressing G06A canine cells were mixed with canine PBMCs, or (C) cPD-L1-expressing HeLa human cells were mixed with human PBMCs, in the presence of the indicated Nb constructs. Cell lysis was recorded 45 min post co-culture for the canine system, or 7 h post co-culture for the human system. (B) Data are mean ± SD calculated from $n = 1$ biological replicate (donor) performed in technical duplicates. (C) Data are ± SD calculated from $n = 3$ biological replicates performed in technical duplicates. Statistical significance was determined using a one-way ANOVA followed by Dunnett's multiple-comparison test, using GraphPad Prism v.10 (*$p < 0.05$ and ****$p < 0.0001$).

drastically improved, they nevertheless exhibited highly stable binding profiles, due to substantially reduced K_{off} values. Importantly, we monitored a striking advantage of all multivalent constructs at interfering with the cPD-1/cPD-L1 signaling pathway, as demonstrated using a newly established cell-based reporter bioassay. We hypothesize that biparatopic and multivalent molecules may efficiently cluster multiple cPD-L1 proteins on the surface of antigen-presenting cells, which consequently may lead to a conformation that is no longer capable to interact with cPD-1 displayed on T cells. Of note, Fc fusions carrying a single or even two different Nbs provide molecules that are still substantially smaller than conventional bivalent antibodies (75–100 vs. 150 KDa), which could translate into better diffusion through tumor tissues and provide further clinical advantages.[35]

Since one of our goals is to develop immune checkpoint therapeutics for the treatment of companion dogs, we chose to equip our constructs with canine IgGA and IgGB isotypes. While the latter is described as able to interact with canine FcγR, the former is not.[28] Thus, IgGB-carrying immune checkpoint inhibitors may additionally result either in the induction of ADCC through interaction with NK cells, or in the triggering of antibody-dependent cell phagocytosis (ADCP) via interaction with monocytes or macrophages. Our data align well with this notion, as ADCC was indeed induced in the presence of Nb constructs containing the dog IgGB Fc domain. Notably, while we employed PBMCs depleted of monocytes and macrophages in the respective assay, we cannot entirely rule out the possibility that ADCP was still triggered to some extent through the interaction of our molecules via FcγRII expressed on any residual monocytes or macrophages. Although most anti PD-(L)1 mAbs developed for human therapy are based on Fc-silent portions, whether it is beneficial to retain Fc binding for their therapeutic profile has been a matter of debate, especially concerning TIGIT,[36] but also PD-L1.[37,38] Moreover, the situation may be even more complex in dogs, since the biology of Fc isotypes is much less studied as compared to humans.[28,39,40] Nevertheless, clinical trials in dogs with spontaneous neoplastic diseases may offer a unique framework to investigate the clinical potential of ADCC/ADCP-triggering vs. silent cPD-L1-targeting immune checkpoint inhibitors.

The integration of an Fc domain into therapeutic drugs can offer several advantages, such as extending the drug half-life in the bloodstream and activating effector functions such as ADCC or ADCP. However, despite being smaller than conventional mAbs, Fc-carrying Nbs may still exhibit reduced tissue penetration compared to monovalent or tandem Nbs. On the other hand, while our Fc-less tandem biparatopic constructs function as highly potent immune checkpoint inhibitors, their clinical potential may be compromised by rapid clearance from the bloodstream. Besides the use of small albumin binders known to extend serum half-life, specific combination therapies with small constructs may provide an alternative solution together with significant advantages for cancer treatment.

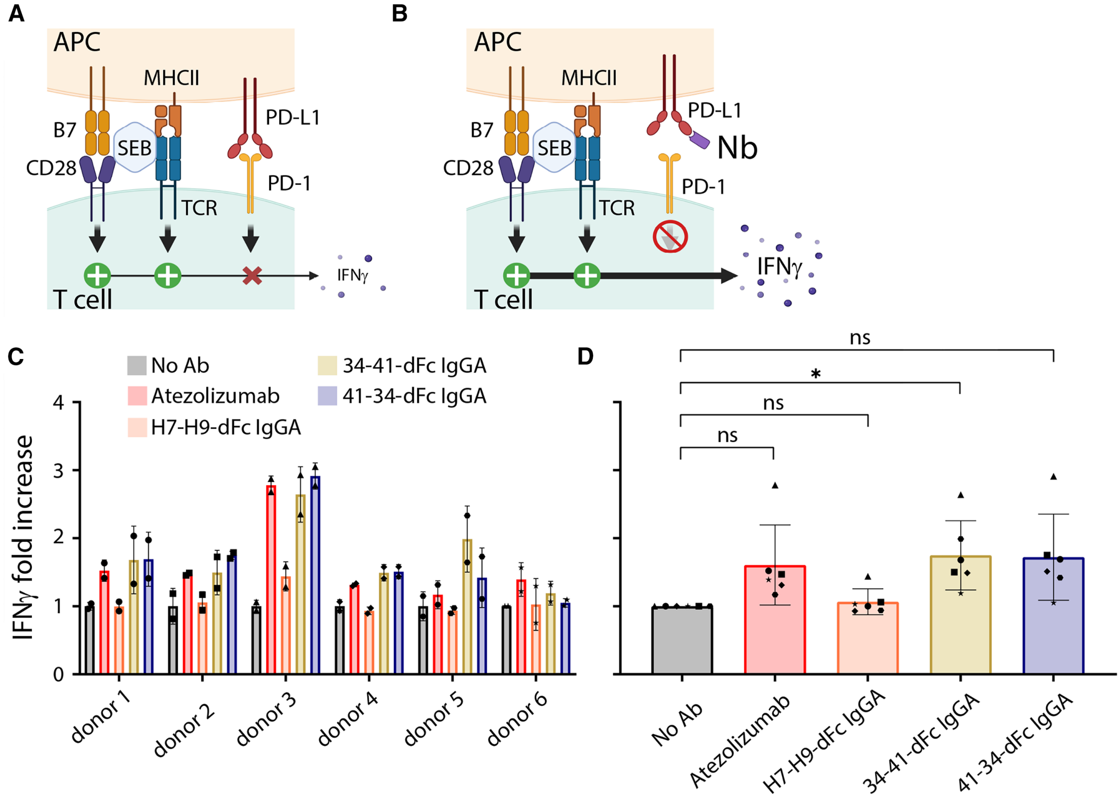

Figure 5. IFNγ production by PBMCs upon treatment with anti-cPD-L1 biparatopic Nb constructs

(A and B) Polyclonal stimulation via SEB leads to IFNγ production, which is increased after blocking of inhibitory PD-1/PD-L1 signaling. (C and D) PBMCs from 6 healthy canine donors were stimulated with SEB (black) or in addition treated with atezolizumab (red), biparatopic Fc fusion nanobodies binding to cPD-L1 (34-41-dFc IgGA and 41-34-dFc IgGA; gold and blue), or a non-binder control (H7-H9-dFc IgGA; orange). Each donor is represented by an individual symbol, fold increase compared to SEB only control shown (C): Data spread *per donor*, mean and technical duplicates shown. (D) Mean ± SD of all donors, calculated *per condition*. Statistical significance was determined using a one-way ANOVA followed by Dunnett's multiple-comparison test, using GraphPad Prism v.10 (*$p < 0.05$).

For instance, oncolytic viruses (OVs) show great promise in cancer therapy,[41] and engineering OVs to deliver Fc-less therapeutics, such as immune checkpoint inhibitors, directly within the tumor microenvironment could lead to much-needed clinical advancements. Beyond the direct lytic effects of OVs on cancer cells, the local secretion of Fc-less therapeutics within neoplastic tissues could not only reduce undesired side effects due to systemic toxicity, but also allow for the use of higher drug concentrations.[35,42] Importantly, OVs may trigger immunogenic cell death, which may in turn facilitate the recruitment of immune cells directly to the tumor site, thereby potentially boosting the clinical impact of the delivered therapeutics.[43]

Although the therapeutic efficacy of our molecules requires further refinements through bioassays using cells isolated from tumors, tumoroids, tumor-infiltrating lymphocytes, or fresh tumor explants,[44] our innovative Nb-based anti-cPD-L1 constructs hold significant potential as valuable tools for gaining deeper insights into drug resistance mechanisms in canine models. Ultimately, Nb-based immunologics, initially tested in companion dogs with spontaneous tumors, may contribute to advancements in cancer therapy for both human and veterinary medicine.

METHODS

Cell lines

The canine G06A cell line derived from spontaneously occurring canine glioblastoma tumors[45] was kindly provided by Dan York and Peter J. Dickinson (The University of California, USA). G06A, HEK-293 (ATCC-CRL-3216), HeLa (ATCC-CCL2), and M113 (gift from CRCINA, Nantes, France) were cultured in Dulbecco's minimal essential Eagle's medium (DMEM, Gibco, BRL, UK) supplemented with 10% heat inactivated fetal bovine serum (Invitrogen/Gibco, Carlsbad, CA, USA) at 37°C and 5% CO_2. G06A and HeLa were transduced with cPD-L1 mRuby2 transgene and were selected under puromycine pression at 1 μg/mL, yielding cell lines HeLa cPD-L1$^+$ and G06A cPD-L1$^+$.

Production and purification of Nbs

After immunization of an alpaca, peripheral blood lymphocytes were collected and phage display library containing genes coding variable

domains of the heavy-chain antibodies (VHH) were generated by Nb platform facility in Zürich. Two rounds of selection on recombinant protein cPD-L1-His (Sino Biological, Beijing, China) were performed following by a screening on the same protein. The phagemid of chosen clones were transformed into BL21 DE3 *E. coli* strain for production of Nbs. The production was induced by culturing the bacteria with isopropyl β-D-1-thiogalactopyranoside (100 μM) at 30°C overnight. The culture was pelleted and lysed in Bugbuster lysis buffer (Merck Millipore, Darmstadt, Germany) supplemented with benzonase (25 U/mL) and lysozyme (20 μg/mL). The His-tagged Nb-containing lysate was charged on TALON superflow (Sigma-Aldrich, Darmstadt, Germany) cobalt resin. After two successive washes in PBS 300 mM NaCl followed by PBS, Nbs were eluted in PBS containing 250 mm of imidazole. Imidazole was removed using PD-10 desalting column (GE Healthcare, Chicago, IL, USA) and Nbs were stored in PBS.

Cytometry binding assay

For cPD-L1 Nbs, HEK293T cells were transiently transfected the day before the experiment with cPD-L1-HA using Lipofectamin3000 (Life Technologies, Carlsbad, CA, USA) following the manufacturer's instruction, using 18 μg of DNA and 30 μL lipofectamin per T75 culture flask. For PD-L1 Nb constructs, stably cPD-L1-expressing G06A or HeLa cells were used. Cells were plated at 200,000 cells per well in 96-well plates and incubated with dilution ranges of purified Nbs or Nb constructs in PBS BSA 1%. A two-step staining was performed using mouse anti-His antibody at 1 μg/mL (Novagen Biotech Labs, Great Neck, NY, USA) followed by goat-anti-mouse Alexa Fluor 647 secondary antibody at 4 μg/mL (Thermo Fisher Scientific, Carlsbad, CA, USA), with three washes in PBS BSA 1% between each step. Cells were analyzed using a MACSQuant X Fast cytometer (Miltenyi Biotec, Bergisch Gladbach, Germany) and affinity was measured as median of fluorescence (MFI) in the R1-A channel (Alexa Fluor 647).

BLI binding assay

The kinetics of Nb and Nb-Fc binding to recombinant cPD-L1-Fc were measured by BLI with a Fortebio Octet R2 instrument (ForteBio, Menlo Park, CA, USA). Briefly, the biotinylated recombinant cPD-L1-Fc (5 μg/mL) were coupled to streptavidin sensors and incubated with a serially diluted Nb or Nb-fusion, followed by dissociation in PBST with BSA buffer. The association rate and dissociation rate were monitored, and the equilibrium K_D was calculated. The data analyses were performed with the Octet analysis studio software and the binding curves were calculated with a 1:1 global fitting model for the Nbs and a 1:2 global fitting model for the Nb-Fc fusion.

In vitro cPD-1/cPD-L1 inhibition assays

cPD-1-Fc (Sino Biological, Beijing, China) was coated overnight at 4°C. After saturation with PBS 2% milk a dilution range of purified Nbs were added for 30 min at RT. Biotinylated cPD-L1-Fc (Sino Biological) was added at 120 nM without washes for an additional hour at RT. Biotinylated PD-L1 Fc binding was revealed with a streptavidin-HRP. The substrate of HRP was added until the colorimetric reaction and stopped with HCl solution. The optic density was measured with a TECAN at 450 nm.

$$\%cPD - \frac{1}{cPDL1} blockade = 100 \times \left(1 - \frac{Absorbance\ 450\ nm_{Nb+cPD-L1}}{Absorbance\ 450\ nm_{cPD-L1\ only}}\right) \quad \text{(Equation 1)}$$

Epitope binning

The epitope binning experiments on recombinant cPD-L1-Fc were performed by BLI with a Fortebio Octet R2 instrument (ForteBio). Briefly, the biotinylated recombinant cPD-L1-Fc (5 μg/mL) were coupled to streptavidin sensors and then incubated with a fixed concentration of Nb1 (100 or 300 nM; depending on their affinity to cPD-L1), followed by second association with a fixed concentration Nb2 (depending on their affinity 100 nM or 300 nM) in the presence of Nb1 at the same concentration as before. The binding values were extracted from Octet analysis studio software.

Production of Nb-fusion

Expi293F cells (Life technologies, Carlsbad, CA, USA) were cultured in Expi293 expression medium in 125 mL flask on orbital shaker at 37°C, 8% CO2. Prior to purification, cells were transiently transfected following the manufacturer's procedure with 30 μg of plasmid for 30 mL culture. The production was performed over 5 days. Fc-carrying constructs were purified on ÄKTA go purification system. Cell-free production supernatants were loaded onto a HiTrap Protein A column (Cytiva, Little Chalfont, United Kingdom). A wash step in PBS was performed, and proteins were eluted using Tris-Glycine (pH 3.5). The purified fraction was neutralized with Tris-HCl pH8 and buffer exchange was performed with PD-10 column to store proteins in PBS. For His-tagged tandems, an IMAC purification was performed using cobalt. Briefly, cell-free supernatant was dialyzed against PBS and purified using TALON resin columns with the same procedure as Nbs. The absence of aggregate was verified by size exclusion chromatography with a Superdex200 increase 10/300 column (Cytiva, Little Chalfont, United Kingdom).

PBMC isolation

Human and canine PBMCs were purified by density gradient centrifugation using Ficoll (Sigma-Aldrich). PBMCs were incubated 24 h for monocyte depletion by plastic adhesion. Peripheral blood from healthy donors was obtained from the French National Blood Bank (Etablissement Français du Sang) using ethically approved procedures. Peripheral blood samples were collected from dog healthy donors at Vet Olympe clinic, Marseille France. For the *ex vivo* IFNγ release assay after basal stimulation with SEB, canine PBMCs were collected from healthy Beagles (according to license no. 299776, ZH242/17, approved by the cantonal veterinary office, Zurich, Switzerland), purified, aliquoted and frozen as previously described.[46]

cPD-1/cPD-L1-induced signaling pathway blockade bioassay

The cPD-1/cPD-L1 blockade bioassay was obtained from Promega. Briefly, cPD-L1-expressing target cells (aAPC/CHO-K1) were seeded in a 96-well white flat bottom plate (Corning, Corning, NY, USA) to a density of 20,000 cells per well. Twenty-four hours later, the medium was removed from the plate and the diluted Nb constructs were added in 40 μL medium. Another 40 μL of cPD-1-expressing Jurkat cells were added. Bio-Glo reagent (80 μL) (Promega, Madison, WI, USA) were added to each well 4 h after the mixture and luminescence was read using a Cytation 5 reader (Biotek, Winooski, VT, USA).

Cytotoxic activity assay by real-time cell analysis

Cytotoxic activities were monitored using xCelligence (Agilent, Santa Clara, CA, USA). cPD-L1-transduced HeLa or G06A cells were seeded in an 8-well plate at 20,000 cells per well and the growing was monitored on RTCA. After 6 h or 24 h of incubation, 100 nM of Nb-constructs and monocytes-depleted PBMC cells were added using an effector/target ratio of 20 or 16, respectively, for human or dog. The cytotoxic activity was monitored for 6 or 20 h by RTCA. The interval slope was calculated automatically by the RTCA software to evaluate the rate of cell index change. To demonstrate the effect of treatments, the cell index was normalized to an equal value at the normalization time point.

The total lysis was determined as follows:

$$\%Total\ Lysis = 100 \times \left(1 - \frac{Normalized\ cell\ index_{cells\ alone}}{Normalized\ cell\ Indes_{cells+PBMC+Ab}}\right) \quad \text{(Equatio 2)}$$

IFNγ release assay

Canine PBMCs from healthy donors were thawed and stimulated as described previously[19]: Briefly, after plating, cells were stimulated with 50 ng/mL SEB (BT202, Toxin Technology, Sarasota, FL, USA) and/or 10 mg/mL atezolizumab (generous gift by H.L., University Hospital Basel, Basel, Switzerland) or 7.6 mg/mL of Nb-Fc constructs (equimolar amounts corresponding to that of 10 mg/mL of atezolizumab) at 37°C for 72 h. At the end of stimulation, the cell culture supernatants were collected and cleared by centrifugation. Concentration of cIFNγ was measured using canine specific ELISA kit (3113-1H-6, Mabtech, Nacka Strand, Sweden) according to the manufacturer's guidelines. Optical density was measured at 450 nm with SPARK plate reader (30086376, Tecan, Männedorf, Switzerland).

Statistical analysis

Data are expressed as the mean ± SD. GraphPad Prism software v.10 (GraphPad Software, San Diego, CA, USA) was used for statistical analysis using a one-way ANOVA followed by Dunnett's multiple-comparison test (*$p < 0.05$ and ****$p < 0.0001$). Regarding the Tables, the mean and SD were obtained using Excel formula AVERAGE and STDEVA on the EC_{50} from triplicates experiments.

DATA AVAILABILITY

The authors confirm that the data supporting the findings of this study are available within the article/supplemental materials. Further inquiries can be directed to the corresponding author.

ACKNOWLEDGMENTS

The authors thank Vet Olympe Marseille, and Elena Lescaudron, for kindly providing dog blood and the owners for their agreement to participate to this study. We also thank Manuela Schnyder, from Institute of Parasitology, Vetsuisse Faculty, University of Zurich, Zurich, Switzerland for kindly providing dog blood. We thank Saša Štefanić from the Nanobody Service Facility of the University of Zurich for his contribution to the immunization campaign, the creation of the library, and the selection of the Nbs, which were essential to the success of this study. We also would like to thank Promega, Jun Wang and Jamison Grailer, for kindly developing and providing the cPD-1/cPD-L1 blocking reporter assay. This work was funded by the Swiss National Science Foundation (Lead Agency: 310030E_189337 to P.P. and P.C.) as well as the international grant ANR PCRi Nanovir (ANR-19-CE18-0036-01 to P.C. and P.P.) and Swiss Cancer Research (grant KFS-5306-02-2021 to J.v.B.).

AUTHOR CONTRIBUTIONS

P.P., P.C., and M.D.P.S. conceived the project. M.D.P.S., M.W., B.T., and M.M. performed experiments. P.P., P.C., and J.v.B. supervised research and obtained funding. M.D.P.S., P.P., and P.C. wrote the first drafts of the paper. All authors reviewed and approved the final manuscript.

DECLARATION OF INTERESTS

J.v.B. has received speaker fees from Bristol Myer Squibb, is a named inventor on patents of the University of Zurich in the field of cancer immunotherapy and a co-founder, shareholder and part-time employee of InCephalo AG. P.C. is a co-founder and shareholder of Nanomunity and Radiomune Pharma. M.D.P.S., M.W., P.C., and P.P. are listed as inventors on a patent application that is related to the subject matter of this manuscript

SUPPLEMENTAL INFORMATION

Supplemental information can be found online at https://doi.org/10.1016/j.omton.2025.201036.

REFERENCES

1. Pardoll, D.M. (2012). The blockade of immune checkpoints in cancer immunotherapy. Nat. Rev. Cancer *12*, 252–264. https://doi.org/10.1038/nrc3239.
2. Okazaki, T., and Honjo, T. (2006). The PD-1-PD-L pathway in immunological tolerance. Trends Immunol. *27*, 195–201. https://doi.org/10.1016/j.it.2006.02.001.
3. Bhatia, S., Edidin, M., Almo, S.C., and Nathenson, S.G. (2005). Different cell surface oligomeric states of B7-1 and B7-2: Implications for signaling. Proc. Natl. Acad. Sci. USA *102*, 15569–15574. https://doi.org/10.1073/pnas.0507257102.
4. Sun, C., Mezzadra, R., and Schumacher, T.N. (2018). Regulation and Function of the PD-L1 Checkpoint. Immunity *48*, 434–452. https://doi.org/10.1016/j.immuni.2018.03.014.
5. Dong, H., Strome, S.E., Salomao, D.R., Tamura, H., Hirano, F., Flies, D.B., Roche, P. C., Lu, J., Zhu, G., Tamada, K., et al. (2002). Tumor-associated B7-H1 promotes T-cell apoptosis: A potential mechanism of immune evasion. Nat. Med. *8*, 793–800. https://doi.org/10.1038/nm730.
6. Zou, W., Wolchok, J.D., and Chen, L. (2016). PD-L1 (B7-H1) and PD-1 Pathway Blockade for Cancer Therapy: Mechanisms, Response Biomarkers and Combinations. Sci. Transl. Med. *8*, 328rv4. https://doi.org/10.1126/scitranslmed.aad7118.
7. Twomey, J.D., and Zhang, B. (2021). Cancer Immunotherapy Update: FDA-Approved Checkpoint Inhibitors and Companion Diagnostics. AAPS J. *23*, 39. https://doi.org/10.1208/s12248-021-00574-0.

8. Sharma, P., Hu-Lieskovan, S., Wargo, J.A., and Ribas, A. (2017). Primary, Adaptive, and Acquired Resistance to Cancer Immunotherapy. Cell *168*, 707–723. https://doi.org/10.1016/j.cell.2017.01.017.

9. Park, J.S., Withers, S.S., Modiano, J.F., Kent, M.S., Chen, M., Luna, J.I., Culp, W.T. N., Sparger, E.E., Rebhun, R.B., Monjazeb, A.M., et al. (2016). Canine cancer immunotherapy studies: linking mouse and human. J. Immunother. Cancer *4*, 97. https://doi.org/10.1186/s40425-016-0200-7.

10. Routy, B., Le Chatelier, E., Derosa, L., Duong, C.P.M., Alou, M.T., Daillère, R., Fluckiger, A., Messaoudene, M., Rauber, C., Roberti, M.P., et al. (2018). Gut microbiome influences efficacy of PD-1–based immunotherapy against epithelial tumors. Science *359*, 91–97. https://doi.org/10.1126/science.aan3706.

11. Song, S.J., Lauber, C., Costello, E.K., Lozupone, C.A., Humphrey, G., Berg-Lyons, D., Caporaso, J.G., Knights, D., Clemente, J.C., Nakielny, S., et al. (2013). Cohabiting family members share microbiota with one another and with their dogs. eLife *2*, e00458. https://doi.org/10.7554/eLife.00458.

12. Kleber, K.T., Iranpur, K.R., Perry, L.M., Cruz, S.M., Razmara, A.M., Culp, W.T.N., Kent, M.S., Eisen, J.A., Rebhun, R.B., and Canter, R.J. (2022). Using the canine microbiome to bridge translation of cancer immunotherapy from pre-clinical murine models to human clinical trials. Front. Immunol. *13*, 983344. https://doi.org/10.3389/fimmu.2022.983344.

13. Igase, M., Nemoto, Y., Itamoto, K., Tani, K., Nakaichi, M., Sakurai, M., Sakai, Y., Noguchi, S., Kato, M., Tsukui, T., and Mizuno, T. (2020). A pilot clinical study of the therapeutic antibody against canine PD-1 for advanced spontaneous cancers in dogs. Sci. Rep. *10*, 18311. https://doi.org/10.1038/s41598-020-75533-4.

14. Maekawa, N., Konnai, S., Okagawa, T., Nishimori, A., Ikebuchi, R., Izumi, Y., Takagi, S., Kagawa, Y., Nakajima, C., Suzuki, Y., et al. (2016). Immunohistochemical Analysis of PD-L1 Expression in Canine Malignant Cancers and PD-1 Expression on Lymphocytes in Canine Oral Melanoma. PLoS One *11*, e0157176. https://doi.org/10.1371/journal.pone.0157176.

15. Hartley, G., Elmslie, R., Dow, S., and Guth, A. (2018). Checkpoint molecule expression by B and T cell lymphomas in dogs. Vet. Comp. Oncol. *16*, 352–360. https://doi.org/10.1111/vco.12386.

16. Stevenson, V.B., Perry, S.N., Todd, M., Huckle, W.R., and LeRoith, T. (2021). PD-1, PD-L1, and PD-L2 Gene Expression and Tumor Infiltrating Lymphocytes in Canine Melanoma. Vet. Pathol. *58*, 692–698. https://doi.org/10.1177/03009858211011939.

17. Shosu, K., Sakurai, M., Inoue, K., Nakagawa, T., Sakai, H., Morimoto, M., Okuda, M., Noguchi, S., and Mizuno, T. (2016). Programmed Cell Death Ligand 1 Expression in Canine Cancer. In Vivo *30*, 195–204.

18. Maekawa, N., Konnai, S., Ikebuchi, R., Okagawa, T., Adachi, M., Takagi, S., Kagawa, Y., Nakajima, C., Suzuki, Y., Murata, S., and Ohashi, K. (2014). Expression of PD-L1 on Canine Tumor Cells and Enhancement of IFN-γ Production from Tumor-Infiltrating Cells by PD-L1 Blockade. PLoS One *9*, e98415. https://doi.org/10.1371/journal.pone.0098415.

19. Pantelyushin, S., Ranninger, E., Guerrera, D., Hutter, G., Maake, C., Markkanen, E., Bettschart-Wolfensberger, R., Rohrer Bley, C., Läubli, H., and Vom Berg, J. (2021). Cross-Reactivity and Functionality of Approved Human Immune Checkpoint Blockers in Dogs. Cancers *13*, 785. https://doi.org/10.3390/cancers13040785.

20. Sirivisoot, S., Boonkrai, C., Wongtangprasert, T., Phakham, T., Muanwein, P., Pisitkun, T., Sawangmake, C., Radtanakatikanon, A., and Rungsipipat, A. (2023). Development and characterization of mouse anti-canine PD-L1 monoclonal antibodies and their expression in canine tumors by immunohistochemistry in vitro. Vet. Q. *43*, 1–9. https://doi.org/10.1080/01652176.2023.2240380.

21. Maekawa, N., Konnai, S., Takagi, S., Kagawa, Y., Okagawa, T., Nishimori, A., Ikebuchi, R., Izumi, Y., Deguchi, T., Nakajima, C., et al. (2017). A canine chimeric monoclonal antibody targeting PD-L1 and its clinical efficacy in canine oral malignant melanoma or undifferentiated sarcoma. Sci. Rep. *7*, 8951. https://doi.org/10.1038/s41598-017-09444-2.

22. Nemoto, Y., Shosu, K., Okuda, M., Noguchi, S., and Mizuno, T. (2018). Development and characterization of monoclonal antibodies against canine PD-1 and PD-L1. Vet. Immunol. Immunopathol. *198*, 19–25. https://doi.org/10.1016/j.vetimm.2018.02.007.

23. Choi, J.W., Withers, S.S., Chang, H., Spanier, J.A., De La Trinidad, V.L., Panesar, H., Fife, B.T., Sciammas, R., Sparger, E.E., Moore, P.F., et al. (2020). Development of canine PD-1/PD-L1 specific monoclonal antibodies and amplification of canine T cell function. PLoS One *15*, e0235518. https://doi.org/10.1371/journal.pone.0235518.

24. Maekawa, N., Konnai, S., Nishimura, M., Kagawa, Y., Takagi, S., Hosoya, K., Ohta, H., Kim, S., Okagawa, T., Izumi, Y., et al. (2021). PD-L1 immunohistochemistry for canine cancers and clinical benefit of anti-PD-L1 antibody in dogs with pulmonary metastatic oral malignant melanoma. npj Precis. Oncol. *5*, 10. https://doi.org/10.1038/s41698-021-00147-6.

25. Oh, W., Kim, A.M.J., Dhawan, D., Kirkham, P.M., Ostafe, R., Franco, J., Aryal, U.K., Carnahan, R.H., Patsekin, V., Robinson, J.P., et al. (2023). Development of an Anti-canine PD-L1 Antibody and Caninized PD-L1 Mouse Model as Translational Research Tools for the Study of Immunotherapy in Humans. Cancer Res. Commun. *3*, 860–873. https://doi.org/10.1158/2767-9764.CRC-22-0468.

26. Yoshimoto, S., Chester, N., Xiong, A., Radaelli, E., Wang, H., Brillantes, M., Gulendran, G., Glassman, P., Siegel, D.L., and Mason, N.J. (2023). Development and pharmacokinetic assessment of a fully canine anti-PD-1 monoclonal antibody for comparative translational research in dogs with spontaneous tumors. mAbs *15*, 2287250. https://doi.org/10.1080/19420862.2023.2287250.

27. Choi, J.W., Withers, S.S., Chang, H., Spanier, J.A., De La Trinidad, V.L., Panesar, H., Fife, B.T., Sciammas, R., Sparger, E.E., Moore, P.F., et al. (2020). Development of canine PD-1/PD-L1 specific monoclonal antibodies and amplification of canine T cell function. PLoS One *15*, e0235518. https://doi.org/10.1371/journal.pone.0235518.

28. Bergeron, L.M., McCandless, E.E., Dunham, S., Dunkle, B., Zhu, Y., Shelly, J., Lightle, S., Gonzales, A., and Bainbridge, G. (2014). Comparative functional characterization of canine IgG subclasses. Vet. Immunol. Immunopathol. *157*, 31–41. https://doi.org/10.1016/j.vetimm.2013.10.018.

29. Pantelyushin, S., Ranninger, E., Guerrera, D., Hutter, G., Maake, C., Markkanen, E., Bettschart-Wolfensberger, R., Rohrer Bley, C., Läubli, H., and Vom Berg, J. (2021). Cross-Reactivity and Functionality of Approved Human Immune Checkpoint Blockers in Dogs. Cancers *13*, 785. https://doi.org/10.3390/cancers13040785.

30. Deng, R., Bumbaca, D., Pastuskovas, C.V., Boswell, C.A., West, D., Cowan, K.J., Chiu, H., McBride, J., Johnson, C., Xin, Y., et al. (2016). Preclinical pharmacokinetics, pharmacodynamics, tissue distribution, and tumor penetration of anti-PD-L1 monoclonal antibody, an immune checkpoint inhibitor. mAbs *8*, 593–603. https://doi.org/10.1080/19420862.2015.1136043.

31. Ahn, J.-H., Lee, B.-H., Kim, S.-E., Kwon, B.-E., Jeong, H., Choi, J.R., Kim, M.J., Park, Y., Kim, B.S., Kim, D.H., and Ko, H.J. (2021). A Novel Anti-PD-L1 Antibody Exhibits Antitumor Effects on Multiple Myeloma in Murine Models via Antibody-Dependent Cellular Cytotoxicity. Biomol. Ther. *29*, 166–174. https://doi.org/10.4062/biomolther.2020.131.

32. Wang, C., Thudium, K.B., Han, M., Wang, X.-T., Huang, H., Feingersh, D., Garcia, C., Wu, Y., Kuhne, M., Srinivasan, M., et al. (2014). *In Vitro* Characterization of the Anti-PD-1 Antibody Nivolumab, BMS-936558, and *In Vivo* Toxicology in Non-Human Primates. Cancer Immunol. Res. *2*, 846–856. https://doi.org/10.1158/2326-6066.CIR-14-0040.

33. Rödström, K.E.J., Elbing, K., and Lindkvist-Petersson, K. (2014). Structure of the superantigen staphylococcal enterotoxin B in complex with TCR and peptide-MHC demonstrates absence of TCR-peptide contacts. J. Immunol. *193*, 1998–2004. https://doi.org/10.4049/jimmunol.1401268.

34. Zimmermann, A.B.E., Taskoparan, B., Fuchs, D., Pantelyushin, S., Maheswaran, M., Schnyder, M., Hartnack, S., Rohrer Bley, C., and Vom Berg, J. (2025). Prior corticosteroid treatment alters cPBMC composition and IFNγ response to immunotherapy in canine cancer. Front. Immunol. *16*, 1544949. https://doi.org/10.3389/fimmu.2025.1544949.

35. Dammes, N., and Peer, D. (2020). Monoclonal antibody-based molecular imaging strategies and theranostic opportunities. Theranostics *10*, 938–955. https://doi.org/10.7150/thno.37443.

36. Dolgin, E. (2020). Antibody engineers seek optimal drug targeting TIGIT checkpoint. Nat. Biotechnol. *38*, 1007–1009. https://doi.org/10.1038/s41587-020-0666-1.

37. Yang, R., Huang, S., Huang, C., Fay, N.S., Wang, Y., Putrevu, S., Wright, K., Zaman, M.S., Cai, W., Huang, B., et al. (2023). Fc-competent multispecific PDL-1/TIGIT/LAG-3 antibodies potentiate superior anti-tumor T cell response. Sci. Rep. *13*, 9865. https://doi.org/10.1038/s41598-023-36942-3.

38. Akinleye, A., and Rasool, Z. (2019). Immune checkpoint inhibitors of PD-L1 as cancer therapeutics. J. Hematol. Oncol. *12*, 92. https://doi.org/10.1186/s13045-019-0779-5.

39. Foltz, J.A., Somanchi, S.S., Yang, Y., Aquino-Lopez, A., Bishop, E.E., and Lee, D.A. (2016). NCR1 Expression Identifies Canine Natural Killer Cell Subsets with Phenotypic Similarity to Human Natural Killer Cells. Front. Immunol. *7*, 521. https://doi.org/10.3389/fimmu.2016.00521.

40. Lee, S.-H., Shin, D.-J., Kim, Y., Kim, C.-J., Lee, J.-J., Yoon, M.S., Uong, T.N.T., Yu, D., Jung, J.-Y., Cho, D., et al. (2018). Comparison of Phenotypic and Functional Characteristics Between Canine Non-B, Non-T Natural Killer Lymphocytes and CD3+CD5dimCD21− Cytotoxic Large Granular Lymphocytes. Front. Immunol. *9*, 841. https://doi.org/10.3389/fimmu.2018.00841.

41. Sivanandam, V., LaRocca, C.J., Chen, N.G., Fong, Y., and Warner, S.G. (2019). Oncolytic Viruses and Immune Checkpoint Inhibition: The Best of Both Worlds. Mol. Ther. Oncolytics *13*, 93–106. https://doi.org/10.1016/j.omto.2019.04.003.

42. Briolay, T., Petithomme, T., Fouet, M., Nguyen-Pham, N., Blanquart, C., and Boisgerault, N. (2021). Delivery of cancer therapies by synthetic and bio-inspired nanovectors. Mol. Cancer *20*, 55. https://doi.org/10.1186/s12943-021-01346-2.

43. Kaufman, H.L., Kohlhapp, F.J., and Zloza, A. (2015). Oncolytic viruses: a new class of immunotherapy drugs. Nat. Rev. Drug Discov. *14*, 642–662. https://doi.org/10.1038/nrd4663.

44. Wu, K.Z., Adine, C., Mitriashkin, A., Aw, B.J.J., Iyer, N.G., and Fong, E.L.S. (2023). Making In Vitro Tumor Models Whole Again. Adv. Healthc. Mater. *12*, 2202279. https://doi.org/10.1002/adhm.202202279.

45. York, D., Higgins, R.J., LeCouteur, R.A., Wolfe, A.N., Grahn, R., Olby, N., Campbell, M., and Dickinson, P.J. (2012). TP53 mutations in canine brain tumors. Vet. Pathol. *49*, 796–801. https://doi.org/10.1177/0300985811424734.

46. Pantelyushin, S., Ranninger, E., Bettschart-Wolfensberger, R., and Vom Berg, J. (2020). OMIP-065: Dog Immunophenotyping and T-Cell Activity Evaluation with a 14-Color Panel. Cytometry, A. *97*, 1024–1027. https://doi.org/10.1002/cyto.a.24168.

OMTON, Volume *33*

Supplemental information

Nanobody-based canine PD-L1-targeting immune checkpoint inhibitors for cancer therapy in dogs

Morgane Di Palma Subran, Marianne Wyss, Betül Taskoparan, Mathischan Maheswaran, Johannes vom Berg, Philippe Plattet, and Patrick Chames

SUPPLEMENTAL MATERIALS AND METHODS

NK cells isolation

Primary human NK cells were isolated from peripheral blood of healthy donors obtained from the French National Blood Bank (Etablissement Français du Sang) using ethically approved procedures. The NK isolation was performed by negative selection using the MACSxpress Whole Blood human NK cell isolation kit (cat. no. 130–098–185, Miltenyi Biotec, Bergisch Gladbach, Germany), according to the manufacturer protocol. Cells were stored in RPMI 1640 medium (Gibco, Life Technologies, Carlsbad, CA, USA) complemented with 10% fetal bovine serum (FBS, Gibco, Life Technologies, Carlsbad, CA, USA) at 37 °C and used in the following 24 h.

The purity of the NK cells was determined by flow cytometry. Isolated primary NK Cells were plated at 200'000 cells per well in 96-well plates and incubated with phenotyping antibodies: anti-CD3-vioblue (cat. 130-094-363), anti-CD56-APC (cat. 130-113-310) and anti-CD16-FITC (cat. 130-113-392) (all from Miltenyi Biotec, Bergisch Gladbach, Germany). After washing, cells were analyzed using a MACSQuant X Fast cytometer (Miltenyi Biotec, Bergisch Gladbach, Germany) and affinity was measured as median of fluorescence (MFI).

Luminescence assay for ADCC measurements

Target cells were plated at 5000 cells/well in 96-well plates (cat. 655083, Greiner, Kremsmünster, Austria) and incubated at 37 °C and 5% CO2 for 6h. Serial dilutions of Nb constructs were added, and cells were incubated for 30 min. Isolated primary NK cells were added (effector:target ratio of 5:1). Cells were further incubated overnight, and the supernatant was rinsed. The cell viability was measured using CellTiter-Glo reagent (Promega, Madison, WI, USA), according to the manufacturer's instructions. The assay reports the amount of ATP from living cells in the sample. The total lysis was determined as followed:

$$\text{Equation S1: } \%Total\ Lysis = 100 \times (1 - \frac{Normalized\ cell\ index_{cells\ alone}}{Normalized\ cell\ Indes_{cells+PBMC+Ab}})$$

SUPPLEMENTAL TABLES

Table S1: Production of the nanobody constructs

Binder	Yield (mg/L)
34-41-hFc IgG1	**248.5**
41-34-hFc IgG1	**252.0**
34-41-dFc IgGB	**161.0**
41-34-dFc IgGB	**161.0**
34-41-dFc IgGA	**37.0**
41-34-dFc IgGA	**48.0**
34-41	**179.5**
41-34	**116.0**
34-hFc IgG1	**600.0**
34-dFc IgGB	**307.0**
34-dFc IgGA	**35.6**
41-hFc IgG1	**849.0**
41-dFc IgGB	**453.0**
41-dFc IgGA	**17.0**

Table S2: Affinity binding of the nanobody constructs by BLI

Binder	KD (M)	KD Error	ka (1/Ms)	ka2	ka Error	ka2 Error	kdis (1/s)	kdis2	kdis Error	kdis2 Error	Full X^2	Full R^2
34-41	**2.21E-08**	4.78E-10	**3.33E05**	9.97E00	**1.98E03**	2.76E00	**7.35E-03**	4.48E-02	**1.53E-04**	1.21E-02	0.05	0.99
34-41	**5.38E-09**	9.44E-11	**2.20E05**	4.72E-02	**1.42E03**	2.31E-03	**1.18E-03**	<1,0E-07	**1.93E-05**	<1,0E-07	0.04	0.99
34-41	**2.51E-08**	7.20E-10	**3.49E05**	2.83E00	**2.34E03**	5.07E-01	**8.76E-03**	1.53E-02	**2.44E-04**	2.54E-03	0.07	0.99
34-41-dFc IgGA	**1.86E-09**	4.99E-11	**2.30E05**	8.46E-02	**1.71E03**	3.83E-03	**4.29E-04**	1.83E-03	**1.10E-05**	3.79E-05	0.17	0.99
34-41-dFc IgGA	**7.16E-09**	7.87E-11	**2.05E05**	3.47E00	**6.39E02**	1.00E00	**1.46E-03**	1.79E-02	**1.54E-05**	4.92E-03	0.15	0.99
34-41-dFc IgGA	**4.95E-09**	6.75E-11	**2.42E05**	4.32E00	**1.20E03**	2.13E-01	**1.20E-03**	2.64E-02	**1.52E-05**	1.44E-03	0.32	0.99
34-41-dFc IgGB	**6.16E-09**	4.77E-11	**2.35E05**	3.63E00	**4.06E02**	2.41E-01	**1.45E-03**	9.09E-03	**1.09E-05**	5.65E-04	0.05	0.99
34-41-dFc IgGB	**5.55E-09**	4.10E-11	**2.42E05**	3.80E00	**4.95E02**	8.50E-02	**1.34E-03**	1.31E-02	**9.53E-06**	3.20E-04	0.06	0.99
34-41-dFc IgGB	**1.32E-09**	2.03E-11	**2.95E05**	5.40E-02	**1.39E03**	1.43E-03	**3.89E-04**	1.25E-03	**5.72E-06**	2.07E-05	0.07	0.99
34-41-hFc IgG1	**1.15E-09**	1.13E-11	**2.91E05**	1.83E-01	**9.22E02**	3.35E-03	**3.35E-04**	5.11E-03	**3.11E-06**	5.33E-05	0.07	0.99
34-41-hFc IgG1	**4.39E-09**	3.39E-11	**3.17E05**	3.89E00	**9.92E02**	1.43E-01	**1.39E-03**	2.72E-02	**9.82E-06**	1.02E-03	0.13	0.99
34-41-hFc IgG1	**5.68E-09**	4.23E-11	**2.81E05**	3.50E00	**5.95E02**	1.48E-01	**1.59E-03**	1.21E-02	**1.14E-05**	4.97E-04	0.07	0.99
34-dFc IgGA	**7.39E-10**	1.58E-11	**1.30E06**	6.78E-01	**5.30E03**	2.85E-02	**9.60E-04**	1.85E-03	**2.01E-05**	2.88E-05	0.01	0.99
34-dFc IgGA	**4.16E-09**	1.67E-10	**2.12E06**	1.97E01	**3.20E04**	1.72E-01	**8.81E-03**	1.94E-02	**3.28E-04**	4.44E-04	0.19	0.96
34-dFc IgGA	**2.51E-09**	3.26E-11	**1.05E06**	1.80E01	**4.75E03**	8.58E-05	**2.63E-03**	1.41E-02	**3.21E-05**	1.52E-04	0.04	0.99
34-dFc IgGB	**4.38E-09**	5.61E-11	**7.54E05**	1.28E01	**3.48E03**	4.50E-01	**3.30E-03**	2.24E-02	**3.95E-05**	8.25E-04	0.04	0.99
34-dFc IgGB	**4.51E-09**	6.59E-11	**5.65E05**	1.63E01	**4.06E03**	5.69E-01	**2.55E-03**	2.25E-02	**3.24E-05**	8.17E-04	0.04	0.99
34-dFc IgGB	**5.68E-09**	8.34E-11	**6.13E05**	1.17E01	**2.53E03**	1.51E00	**3.48E-03**	1.29E-02	**4.91E-05**	1.59E-03	0.03	0.99
34-hFc IgG1	**2.57E-09**	4.14E-11	**7.41E05**	1.24E01	**5.94E03**	4.17E00	**1.90E-03**	4.36E-02	**2.66E-05**	1.40E-02	0.08	0.99
34-hFc IgG1	**2.32E-09**	3.15E-11	**6.40E05**	2.01E01	**4.95E03**	1.22E-01	**1.49E-03**	4.66E-02	**1.66E-05**	1.13E-03	0.05	0.99
34-hFc IgG1	**4.70E-09**	9.19E-11	**9.34E05**	9.09E00	**5.16E03**	7.59E-01	**4.40E-03**	1.23E-02	**8.24E-05**	1.00E-03	0.10	0.99
41-34	**3.24E-08**	9.99E-10	**3.22E05**	1.01E01	**1.71E03**	4.17E00	**1.04E-02**	2.18E-02	**3.17E-04**	8.76E-03	0.04	0.99
41-34	**1.85E-08**	4.62E-10	**5.31E05**	9.48E00	**3.46E03**	4.05E00	**9.83E-03**	4.467E-02	**2.37E-04**	1.87E-02	0.07	0.99
41-34	**4.28E-09**	6.89E-11	**3.12E05**	4.95E-02	**1.89E03**	2.29E-03	**1.34E-03**	<1,0E-07	**1.99E-05**	<1,0E-07	0.06	0.99
41-34-dFc IgGA	**1.15E-09**	2.52E-11	**4.22E05**	1.04E-01	**2.99E03**	4.15E-03	**4.87E-04**	2.08E-03	**1.00E-05**	4.33E-05	0.21	0.99
41-34-dFc IgGA	**8.33E-09**	1.04E-10	**2.583E05**	3.197E00	**7.56E02**	8.03E-01	**2.15E-03**	1.11E-02	**2.62E-05**	2.63E-03	0.16	0.99

41-34-dFc IgGA	**4.43E-09**	6.14E-11	**3.22E05**	4.40E00	**1.46E03**	2.02E-01	**1.42E-03**	1.52E-02	**1.86E-05**	7.77E-04	0.31	0.99
41-34-dFc IgGB	**3.95E-09**	3.49E-11	**3.28E05**	2.66E00	**5.67E02**	3.15E-01	**1.30E-03**	5.48E-03	**1.12E-05**	5.57E-04	0.05	0.99
41-34-dFc IgGB	**4.52E-09**	2.03E-11	**2.92E05**	6.40E00	**3.25E02**	9.38E-04	**1.32E-03**	1.00E-02	**5.72E-06**	4.77E-05	0.06	0.99
41-34-dFc IgGB	**4.47E-09**	4.05E-11	**3.12E05**	6.16E00	**5.72E02**	6.97E-03	**1.40E-03**	5.57E-03	**1.24E-05**	2.91E-05	0.06	0.99
41-34-hFc IgG1	**2.34E-09**	2.02E-11	**4.04E05**	2.09E00	**1.01E03**	2.27E-01	**9.32E-04**	6.99E-03	**7.84E-06**	6.24E-04	0.09	0.99
41-34-hFc IgG1	**3.66E-09**	3.06E-11	**3.38E05**	6.24E00	**9.45E02**	2.59E-02	**1.24E-03**	1.44E-02	**9.74E-06**	1.99E-04	0.07	0.99
41-34-hFc IgG1	**2.81E-09**	1.87E-11	**3.79E05**	6.15E00	**5.34E02**	3.03E-02	**1.07E-03**	9.80E-03	**6.92E-06**	7.14E-05	0.04	0.99
41-dFc IgGA	**8.81E-09**	8.16E-10	**3.25E04**	9.14E-03	**1.23E03**	2.12E-03	**2.87E-04**	<1,0E-07	**2.41E-05**	<1,0E-07	0.11	0.99
41-dFc IgGA	**1.70E-09**	9.11E-11	**5.83E04**	3.30E-08	**3.67E02**	1.76E01	**9.92E-05**	2.78E00	**5.28E-06**	1.48E09	0.01	0.99
41-dFc IgGA	**7.03E-09**	2.48E-10	**4.57E04**	3.78E-03	**8.21E02**	8.39E-04	**3.21E-04**	<1,0E-07	**9.77E-06**	<1,0E-07	0.09	0.99
41-dFc IgGB	**5.84E-09**	1.49E-10	**2.48E05**	1.41E00	**1.54E03**	3.05E-01	**1.45E-03**	6.41E-03	**3.58E-05**	1.10E-03	0.24	0.99
41-dFc IgGB	**4.57E-09**	1.11E-10	**2.08E05**	1.30E00	**7.18E02**	1.92E-01	**9.52E-04**	2.96E-03	**2.29E-05**	2.89E-04	0.06	0.99
41-dFc IgGB	**2.57E-09**	7.78E-11	**2.10E05**	6.44E-02	**1.87E03**	3.40E-03	**5.40E-04**	1.13E-03	**1.56E-05**	3.96E-05	0.16	0.9
41-hFc IgG1	**1.40E-09**	2.54E-11	**7.50E05**	1.17E00	**5.29E03**	8.92E-02	**1.05E-03**	6.63E-03	**1.76E-05**	3.12E-04	0.34	0.99
41-hFc IgG1	**1.18E-09**	4.40E-11	**4.26E05**	7.77E-02	**7.15E03**	5.50E-03	**5.01E-04**	2.47E-03	**1.68E-05**	1.43E-04	0.36	0.99
41-hFc IgG1	**2.87E-09**	7.02E-11	**1.95E05**	6.05E-02	**1.35E03**	2.58E-03	**5.60E-04**	1.10E-03	**1.31E-05**	3.28E-05	0.11	0.99

Table S3: Characteristics of healthy PBMC dog donors

#	SEX	Age (years)	Breed	ASSAY
**0**	Female	5	Maltese	ADCC
**1**	Male	2	Beagle	cIFNγ production
**2**	Female	2	Beagle	cIFNγ production
**3**	Male	2	Beagle	cIFNγ production
**4**	Male	2	Beagle	cIFNγ production
**5**	Female	2	Beagle	cIFNγ production
**6**	Female	2	Beagle	cIFNγ production

SUPPLEMENTAL FIGURES & FIGURES LEGENDS

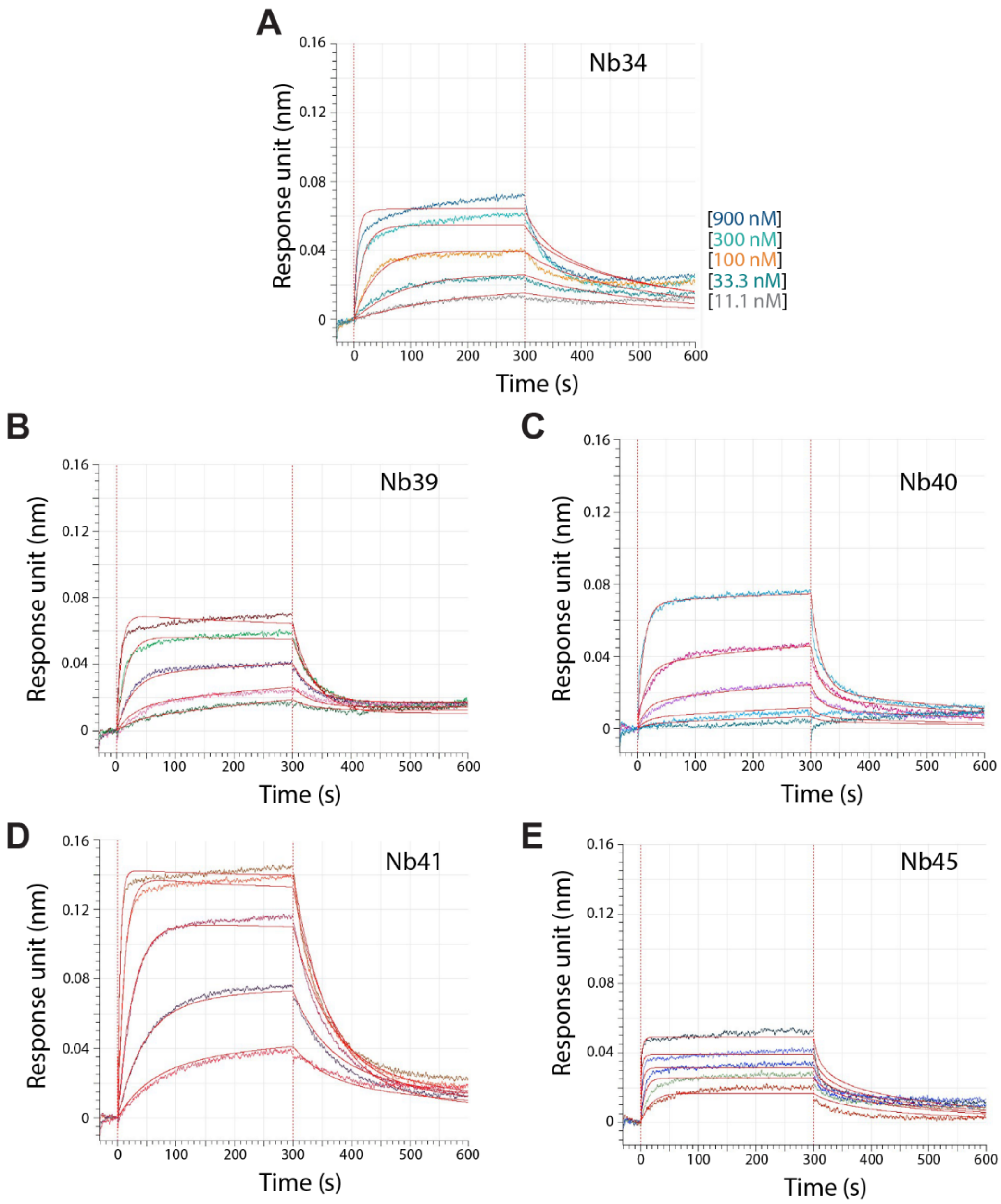

Figure S1: Binding of the anti-cPD-L1 nanobodies to recombinant cPD-L1 ectodomain

(**A-E**) Binding activity of the indicated Nbs against the immobilized cPD-L1 ectodomain were analyzed by BLI. Raw data are color-coded according to the assessed Nb-concentration. Fitted curves are plotted as red lines employing the Octet analysis studio software®. Vertical dotted red lines correspond to either the transition between the base line and the association phase (left) or the transition between the association and dissociation phases (right). Data were collected from *n* = 3 independent experiments using the Octet®R2 device. One representative experiment is displayed.

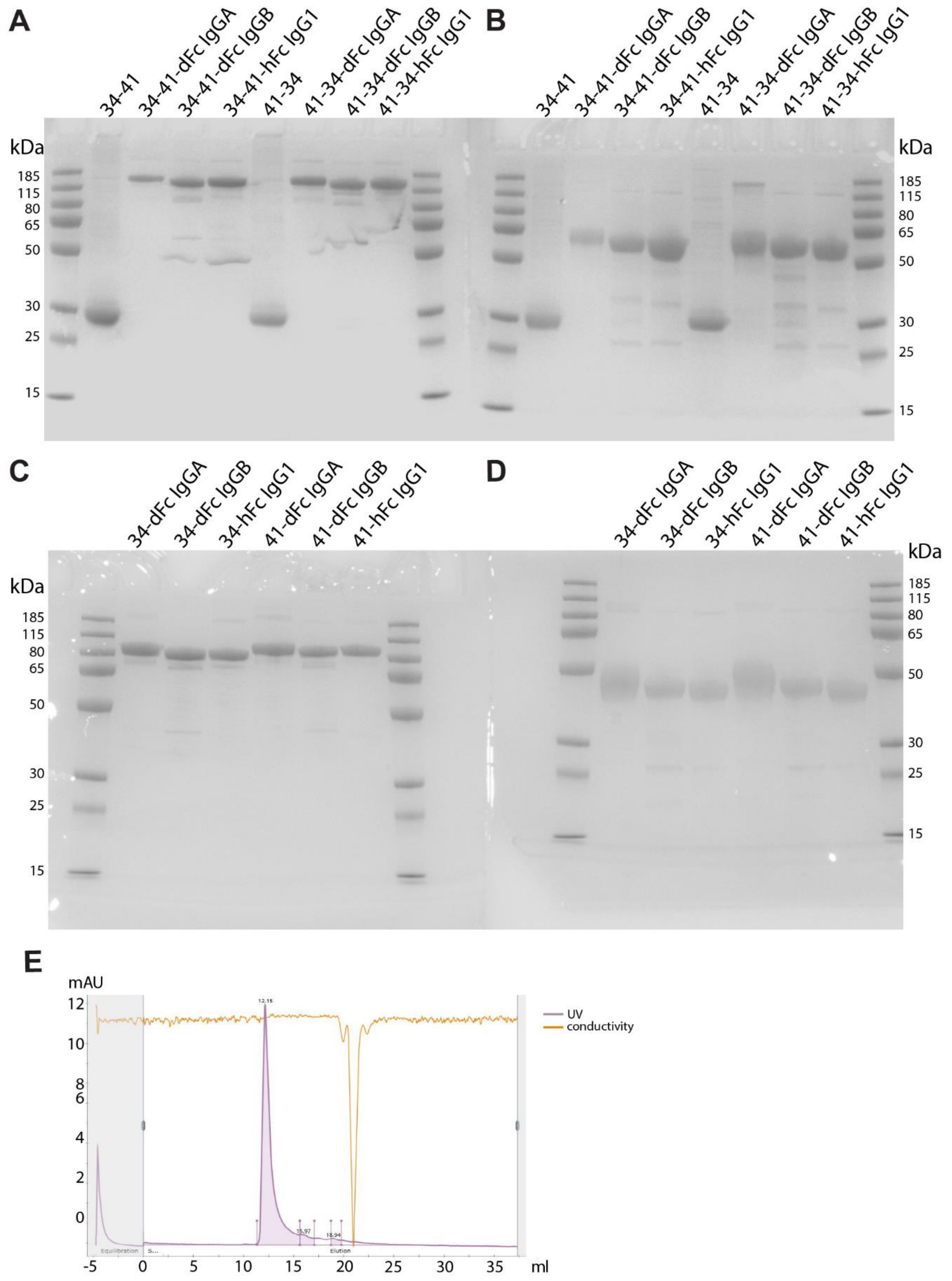

Figure S2: Biochemical characterization of Nb constructs.

Nb constructs were produced in mammalian cells and their molecular weight was confirmed by SDS-PAGE analyses ran under reducing (**B and D**) and nonreducing (**A and C**) conditions. (**E**) The purity of the Nb constructs was assessed by Size Exclusion Chromatography (SEC) using a

Superdex 200 increase 10/300 GL (Cytivia). One representative experiment is displayed with Nb 34-41-dFc IgGB.

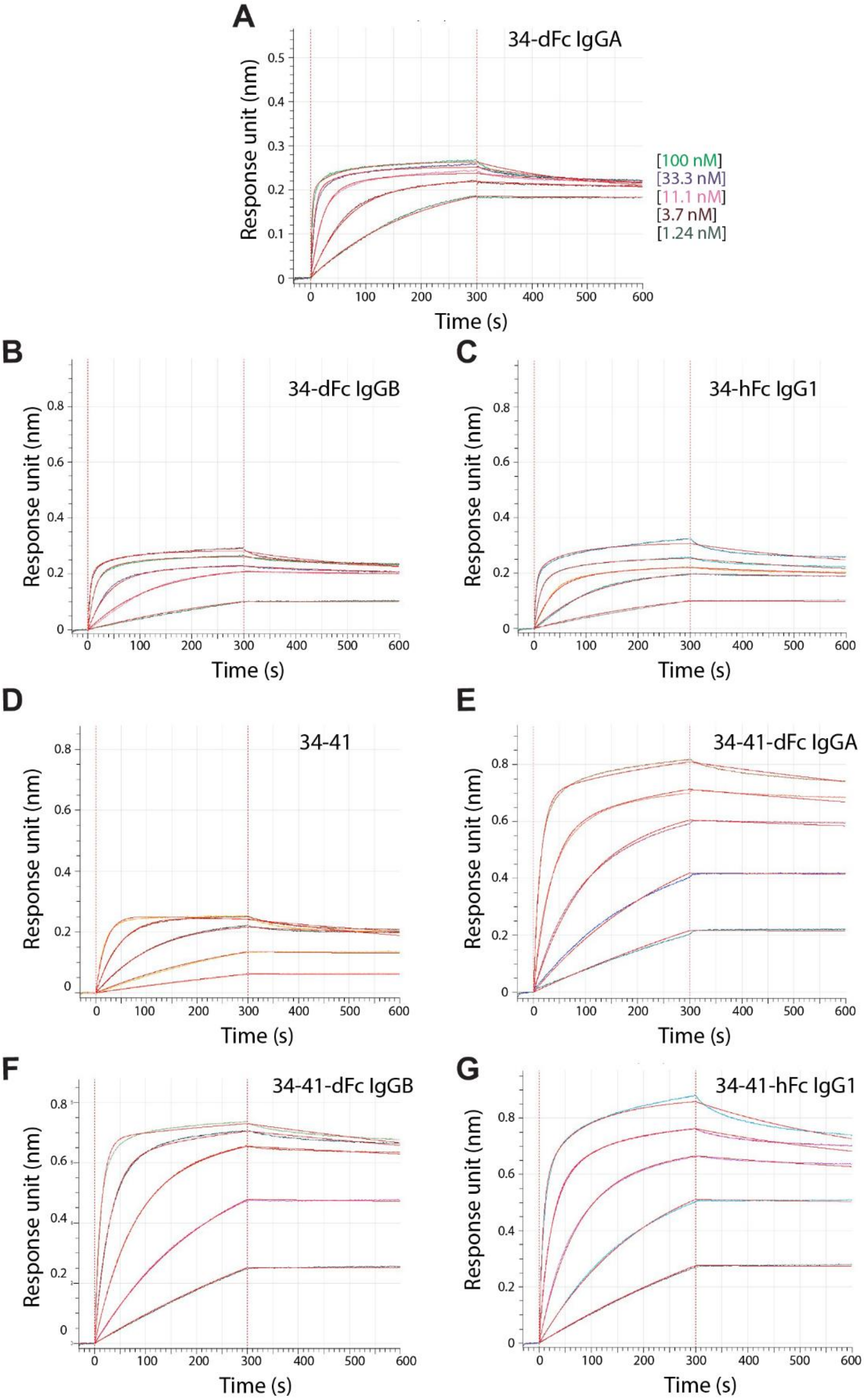

Figure S3: Binding of the Nb34-derived constructs to recombinant cPD-L1 ectodomain.

(**A-G**) Binding activity of the indicated Nbs against the immobilized cPD-L1 ectodomain were analyzed by BLI. Raw data are color-coded according to the assessed Nb-concentration (indicated on the right of panel (A)). Fitted curves are plotted as red lines employing the Octet

analysis studio software®. Vertical dotted red lines correspond to either the transition between the base line and the association phase (left) or the transition between the association and dissociation phases (right). Data were collected from n = 3 independent experiments using the Octet®R2 device. One representative experiment is displayed.

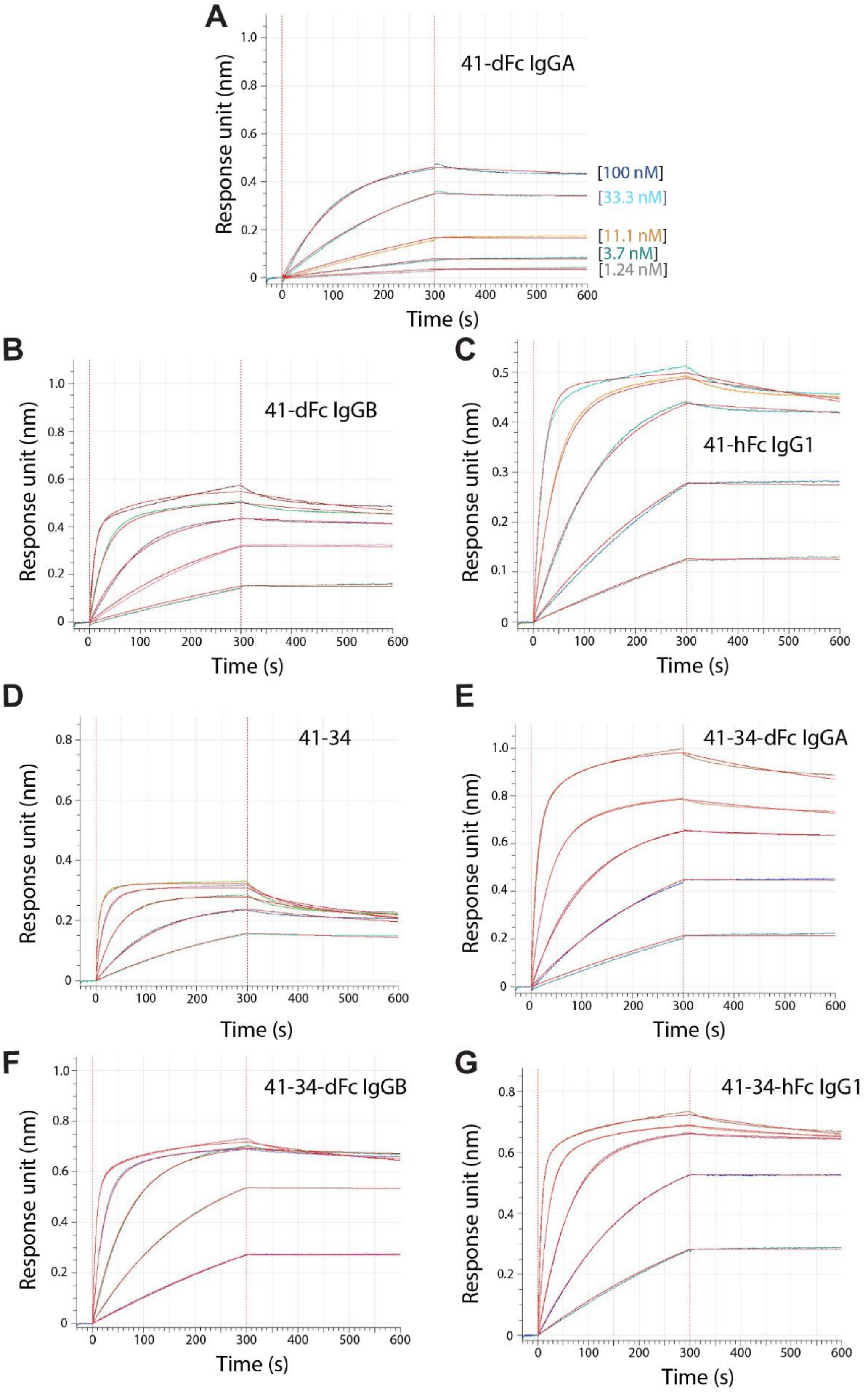

Figure S4: Binding of the Nb41-derived constructs to recombinant cPD-L1 ectodomain.

(**A-G**) Binding activity of the indicated Nbs against the immobilized cPD-L1 ectodomain were analyzed by BLI. Raw data are color-coded according to the assessed Nb-concentration (indicated on the right of panel (A)). Fitted curves are plotted as red lines employing the Octet analysis studio software®. Vertical dotted red lines correspond to either the transition between the base line and the association phase (left) or the transition between the association and dissociation phases (right). Data were collected from n = 3 independent experiments using the Octet®R2 device. One representative experiment is displayed.

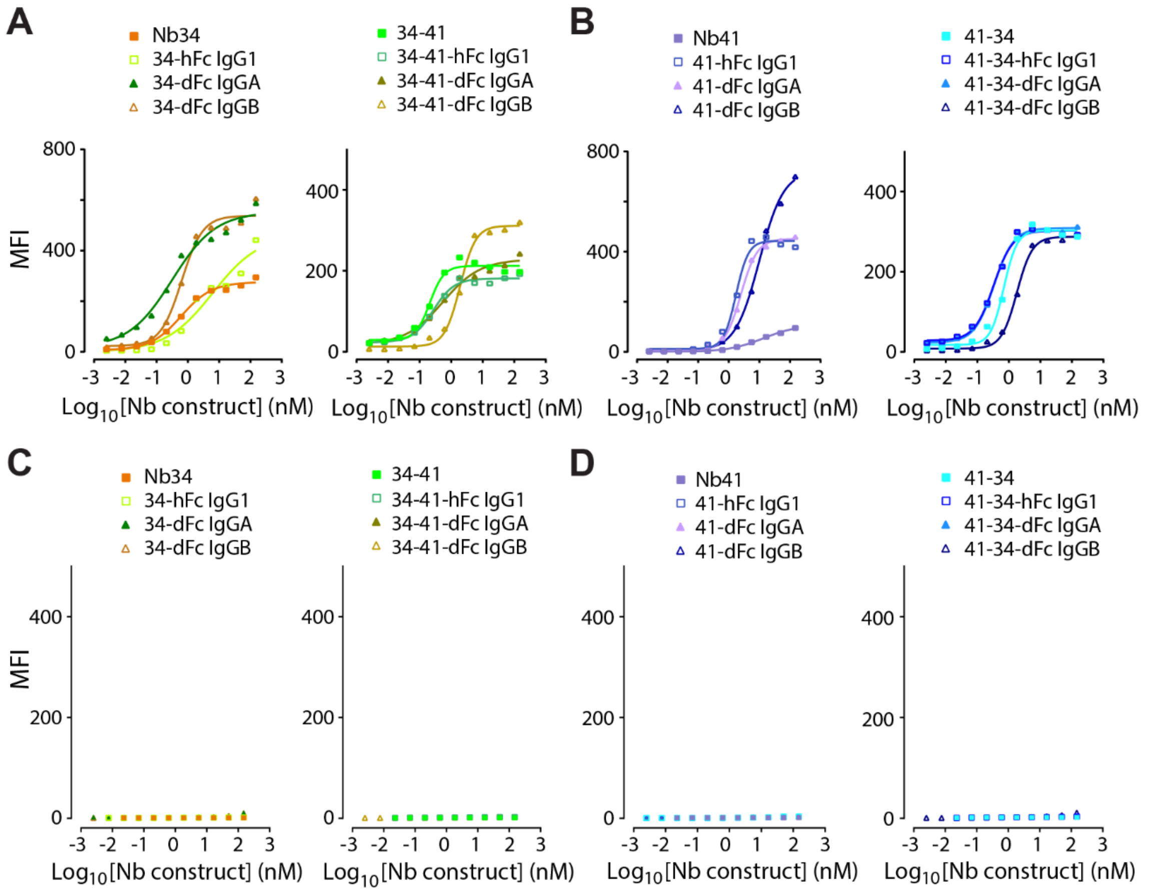

Figure S5: Binding of the Nbs and derivatives constructs to membrane-anchored cPD-L1.

Assessment of the binding efficiency of the Nbs and derivative constructs on cPD-L1-expressing HeLa (**A and B**) and (cPD-L1-untransduced) G06A (**C and D**) cells by flow cytometry. Binding was detected with a mouse anti-HIS followed by an Alexa eFluor647-conjugated goat anti-mouse IgG. Binding assessment was obtained from n = 3 independent experiments. One representative experiment is shown. MFI data were extracted from single cells gating using Flowlogic software™ and curves were plotted with GraphPad Prism v.10.

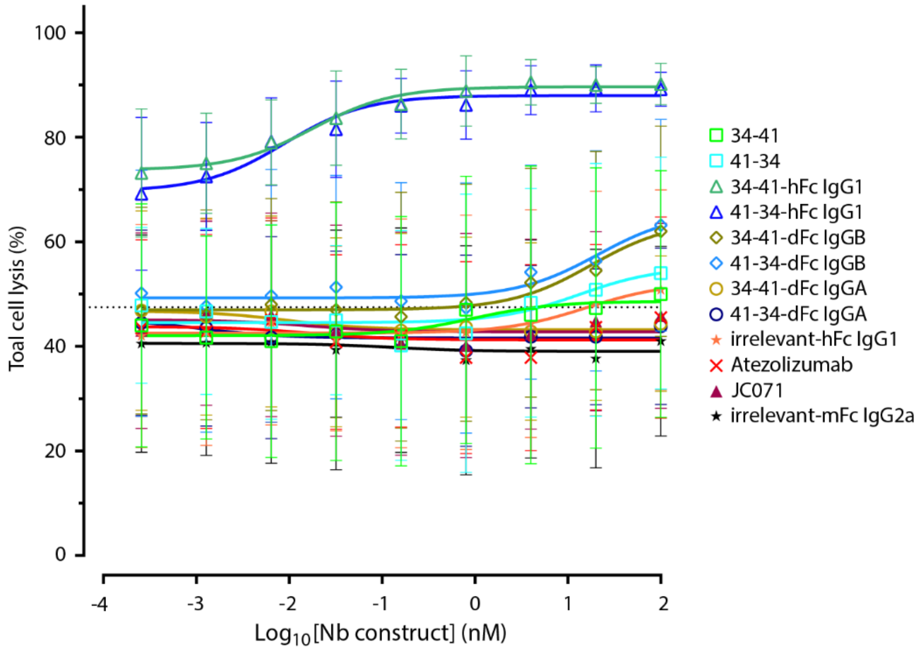

Figure S6: Assessment of the ADCC-inducing ability of the Nb constructs.

ADCC-triggering by the nanobody constructs was determined by employing canine PD-L1-expressing HeLa human cells co-cultured with purified human NK cells in the presence of Fc-carrying (or not) anti-canine PD-L1 Nb constructs. Cell lysis, induced by Fc-dependent activation of CD16-expressing NK cells, was recorded after 16h of incubation using Cell titer glo kit (Promega). Data are mean ± SD calculated from n = 3 biological replicate (donor) performed in technical duplicates.
